# Sodium aescinate inhibits microglia activation through NF-κB pathway and exerts neuroprotective effect

**DOI:** 10.3389/fphar.2023.1086429

**Published:** 2023-01-26

**Authors:** Fei Xu, Yiguo Jiang, Xiaoyu Wang, Li Shen, Yan Yan, Dongkai Guo, Cheng Wang

**Affiliations:** ^1^ Department of Pharmacy, Suzhou Science and Technology Town Hospital, Suzhou, China; ^2^ Department of Pharmacy, The People’s Hospital of Suzhou New District, Suzhou, China; ^3^ Department of Pharmacy, The Affiliated Suzhou Hospital of Nanjing Medical University, Suzhou Municipal Hospital, Suzhou, China; ^4^ Department of Neurology, Suzhou Science and Technology Town Hospital, Suzhou, China; ^5^ High-tech Zone social utilities bureau of Suzhou, Suzhou, China

**Keywords:** sodium aescinate, microglia, NF-κB pathway, neuroinflammation, molecular docking

## Abstract

**Background:** Microglia are resident immune cells of the central nervous system that sense environmental changes and maintain central nervous system homeostasis. Dysfunctional microglia produce toxic mediators that lead to neuronal death. Recent studies suggest that Sodium Aescinate has a neuroprotective effect. However, it is unclear whether Sodium Aescinate exerts neuroprotective effects by inhibiting activation of microglia.

**Method:** Traumatic brain injury and lipopolysaccharide neuroinflammation model were used to evaluate the microglia activation *in vivo*. BV2 and primary microglia cells were used to assess the microglia activation *in vitro*. Molecular docking technique was used to predict the binding energy of Sodium Aescinate to NF-κB signaling pathway proteins.

**Result:** Sodium Aescinate inhibited microglial activation *in-vivo* and *in-vitro*. Sodium Aescinate inhibited the activation of microglia in Traumatic brain injury and lipopolysaccharide mouse models. Sodium Aescinate also inhibited the expression of inflammatory proteins in BV2 and primary microglia cells. Western blot experiment showed that SA inhibited the activation of NF-κB pathway in BV2 and primary microglia cells. Molecular docking results also showed that Sodium Aescinate had a better affinity with the core protein of the NF-κB pathway. Western blot identified that SA inhibited activation of NF-κB pathway. In Traumatic brain injury model and conditioned medium experiment, Sodium Aescinate pretreatment inhibited inflammation and protected neuron.

**Conclusion:** Our study confirmed that the protection effects of Sodium Aescinate on neurons by inhibiting microglia activation through NF-κB pathway.

## Introduction

Microglia are resident neuroimmune cells of the central nervous system, which sense environmental changes, remove microorganisms, protein aggregates, particles, soluble antigens, and maintain central nervous homeostasis (Amor et al., 2022; Spiteri et al., 2022). Activated microglia released inflammatory cytokines such as TNF-α and IL-6 and reactive oxygen species (ROS) to damage neurons (Simpson and Oliver, 2020). Traumatic brain injury (TBI) is an aseptic injury in which external forces act on the head. TBI causes a high disability rate and mortality, results in serious social and economic burden, and attracts widespread social concerns (Jiang et al., 2019). Microglia was activated after TBI, leading to secondary brain injury, which has complex pathological and pathogenic mechanisms (Willis et al., 2020; Somebang et al., 2021). Toll-like receptors (TLRs) is a member of pattern recognition receptors (PRR) expressed on the surface or inside of innate immune cells. LPS can specifically activate TLR4 expressed on the surface of microglia and is commonly used as an activator of NF-κB signaling pathway (Zusso et al., 2019). Therefore, inhibition of microglial activation *via* NF-κB signaling pathway is a promising therapeutic approach for neuroinflammation.

Aescin, a triterpenoid saponin containing ester bonds, is extracted from the dried and mature seeds of Aesculus or Chestnut (Huang et al., 2022). Sodium Aescinate (SA) has a phenolic hydroxyl structure, which timely and effectively scavenges oxygen free radicals and plays a neuroprotective role (Wang et al., 2012). Recently, SA was reported that it reduced oxidative stress and provided neuroprotection in experimental traumatic brain and spinal cord injury (Cheng et al., 2016; Zhang et al., 2020). However, it is unclear whether SA exerts neuroprotective effects by inhibiting activation of microglia.

## Materials and methods

### Materials

SA was purchased from Selleck Company, the chemical formula is C_54_H_83_NaO_23_ and the molecular weight is 1,123.21.5 mg. SA was accurately weighed and 89.03 µL DMSO was added to prepare 50 mM mother liquor, which was stored in a refrigerator at −20°C and diluted with PBS solution to the desired concentration before use. The choice of SA dose (1 mg/kg) in mice was based on the reference (Zhang et al., 2020).

### Extraction of primary microglia

C57BL/6 neonatal mouse born 1–2 days old were sterilized in 75% alcohol. The mouse brain was removed and cut into pieces before the meninges were peeled off. The minced mouse brain was placed in a centrifuge tube containing 0.25% trypsin (Gibco United States) and maintained at 37°C for about 15 min. The minced mouse brain was centrifuged and the supernatant was discarded, washed twice with DMEM. Next DNase was added to 100 μg/mL, and incubated at room temperature for 1–2 min. The compound was centrifuge briefly to discard the supernatant and DMEM medium was used to resuspend the pellet which pass through a 40 µm filter next. The primary cells in filtrate were put into a PDL-coated culture bottle then added appropriate amount of medium in bottle. The medium was changed after 1 day of culture, then changed every 3 days. The primary cells were cultured for 7–14 days and separated. The bottle was sealed with parafilm and placed in 250 rpm and 37°C shaker bed for 2 h. The primary microglia cells in the supernatant were collected in PDL Coated Bottles.

### Cell culture

The BV2 cell lines were presented from Wang lab research group, School of Pharmacy, Soochow University. BV2 cells and primary microglia were cultured in DMEM medium (Gibco United States) containing 10% fetal bovine serum (Gibco United States), 100 μg/mL penicillin and 100 μg/mL streptomycin. The human neuroblastoma cell lines SH-SY5Y were cultured in DMEM/F12 medium (Gibco United States) containing 10% fetal bovine serum, 100 μg/mL penicillin and 100 μg/mL streptomycin in a humidified incubator with 5% CO_2_ at 37°C.

### Animals

Male C57BL/6 mice (18–22 g) were purchased from Shanghai Slack Laboratory Animal Co., Ltd., Each group was comprised of six mice. All the mice were reared under controlled temperature (22°C ± 1°C) and humidity (50 ± 5%) conditions in a 12-h light/dark cycle with food and water available *ad libitum*. All experimental procedures were approved by the Animal Care and Use Committee of Suzhou Institute of Medical Technology (Suzhou, China). Each group was comprised of six mice (Ethics Number: 2022-B19).

### TBI animal model

Mice were anesthetized using 50 mg/kg pentobarbital sodium. Then the surgical area of mouse head was wiped with alcohol, the scalp was cut about 2 cm along the middle to the right slightly with scissors, soft tissue and bony membrane were separated bluntly. The skull was exposed. A circular bone window with a diameter of 4 mm was opened by a skull drill in front of the herringbone suture about 2 cm and beside the midline of the skull about 2 mm. A weight of 5 g was dropped vertically from a height of 15 cm above the piston in the condition of dura mater intact, which was perpendicular to the bone window and the bottom of the piston matched the size of the bone window. The brain was injured by striking the piston. In the control group, only the bone window was opened without injury. The mice were placed on the thermal insulated blanket before suturing the scalp and returned to the cage after awakening.

### LPS animal model

Mice were anesthetized using 50 mg/kg pentobarbital sodium. Then mice proned on the fixator of the stereotaxic instrument (RWD Life Science Co., China). The ear sticks on both sides of the localizer were inserted into both ears of the mouse with the distance about 7 mm. The scalp of the mouse was wiped with alcohol and cut along the sagittal plane with scissors. The periosteum of the skull cap was scraped off with a blade to expose the skull bone fully. The chimney point, also named Bregma point, was found in the junction of the coronal suture (herringbone suture) and sagittal suture. A small hole was drilled with a normal syringe needle at 3.5 mm posterior and 1.2 mm left/right of the point of Bregma. The syringe needle just penetrated the skull so that the microsyringe just passed through. The needle of microsyringe decreased by 4.5 mm, then the drug was slowly injected into the mouse brain at a rate of 0.1 μL/min through the control of the micro-injection pump. After the end of injection, the drug was kept still for 3 min to allow the drug to be completely absorbed, then the needle was slowly pulled out to avoid bleeding. The mice were placed on the thermal insulated blanket before suturing the scalp and returned to the cage after awakening.

### Immunostaining

Mice brains were removed and then soaked in 4% PFA at 4°C overnight, then dehydrated with a sucrose gradient of 20% and 30% respectively. Mice brains were cut into brain slices with a thickness of 20 µm with a cryostat (Leica, Germany). Brains and primary microglia cell were fixed with 4% PFA, washed with PBS, punched with 0.25% TritonX-100 and blocked with 4% BSA. Brains and primary microglia cell were stained by IBA1 primary antibody (Wako, Japan) and placed in a refrigerator at 4°C overnight. The brain slices were stained by fluorescence anti-rabbit/mouse secondary antibody (Invitrogen, United States), transferred to glass slides and mounted with DAPI-containing (Abcam, United Kingdom) mounting medium. Finally, brain slices were observed and photographed under an inverted fluorescence microscope (Olympus, Japan). Primary microglia cells were stained by DAPI (vector, United States) for nucleus. The state and purity of primary microglia were observed and photographed under an upright fluorescence microscope (Olympus, Japan).

### NO assay

BV2 cells were seeded in 96-well plates at a cell density of 5×10^4^ cells/mL overnight. Cells were pretreated by SA (5 µM) for 12 h, then treated by LPS (100 ng/mL) for 12 h, Three replicate wells were prepared. 50 µL of the supernatant from different treatments were transferred into a new 96-well plate. According to the instructions of NO kit (Beyotime, china), 50 µL/well of Griess Reagent I was added, followed by 50 µL/well of Griess Reagent II. The absorbance was measured at a wavelength of 540 nm using a microplate reader (Tecan, Switzerland).

### ROS assay

BV2 Cells were seeded in 12-well plates with a cell density of 1 × 10^6^ cells/mL overnight. Cells were treated with SA (5 µM) and LPS (100 ng/mL) accordingly. According to the ROS kit (Beyotime, China), the original culture medium in the culture dish was discarded and the cells were washed three times with preheated serum-free medium and loaded probe DCFH-DA at a concentration of 10 μm/L (the solvent was serum-free medium), the cells were incubated in a 37°C cell culture incubator for 20 min. After washed with serum-free medium, the cells were collected and detected by flow cytometry (NovoCyte, Canada).

### CCK-8 assay

BV2 Cells were seeded in 96-well plates at a cell density of 5 × 10^4^ cells/mL overnight. Cells were pretreated by different concentrations of SA (200, 100, 50, 25, 10, 5, 1, 0 µM) for 24 h in a 37°C incubator, with three replicate wells for each concentration. Then CCK-8 detection reagent (biosharp, China) was added in 96-well plates according to the standard protocol in the kit instructions, and the cells were continued cultured in the incubator for approximately 2 h. Then the absorbance values were measured at a wavelength of 450 nm using a microplate reader and statistically analyzed.

### Western blot assay

Protein was extracted from tissues or cells using RIPA lysis solution (Beyotime Biotechnology, China) containing protease inhibitors (Beyotime, China). Protein quantification was performed with BCA protein quantification kit (Beyotime Biotechnology, China), approximately 30 μg of protein was separated on 8%–12% SDS-polyacrylamide gel. Isolated protein was transferred to a PVDF membrane (Millipore, United States)which was incubated with primary antibody iNOS (1:1,000 dilution, Abcam, United Kingdom) COX2 (1:2000 dilution, CST, United States) p-P65 (1:2,000 dilution, CST, United States) p-IκK (1:1,000 dilution, CST, United States) p-IκK (1:1,000 dilution, CST, USA) IκB (1:1,000 dilution, CST, United States) IκB (1:1,000 dilution, CST, United States) GAPDH (1:5,000 dilution, Santa Cruz Biotechnology, United States) Caspase3 (1:1,000 dilution, CST, United States) overnight at 4°C in a refrigerator. Anti-rabbit horseradish peroxide-conjugated antibody (1:5,000 dilution, BBI, Canada) or anti-mouse horseradish peroxide-conjugated antibody (1:5,000 dilution, BBI, Canada) was used as the secondary antibody. The blots were incubated with ECL chemiluminescence reagent (Clinx, China), and the expression of protein was detected with chemiluminescence imaging analysis system (Clinx, China). Quantification was performed with ImageJ software.

### ELISA for cytokines secretion

BV2 cells were cultivated in 12-well plates at a density of 2 × 10^5^ cells/mL with 1 mL medium per well and treated with LPS and desired concentrations of SA for 24 h. The supernatant of BV2 cells treated by LPS or SA was detected by IL-6 and TNF-α mouse ELISA kit (BOSTER, China) according to the manufacturer’s instructions. The OD value was measured by a microplate reader at 450 nm and the sample concentration was calculated by the linear regression equation established by the standard concentration and OD value.

### Quantitative real-time PCR (qRT-PCR)

Total RNA was extracted from BV2 cells using TRIzol reagent (Invitrogen, United States), cDNA was reversely transcribed from total RNA using PrimeScript RT Master Mix (Takara, Japan), qRT-PCR analysis was performed using the 2^−ΔΔCT^ method. Primers for qRT-PCR were designed as follows: Mouse iNOS: forward primer: 5-TCC​CAG​CCT​GCC​CCT​TCA​AT-3 and reverse primer: 5-CGG​ATC​TCT​CTC​CTC​CTG​GG-3; COX-2: forward primer: 5-CAG​GCT​GAA​CTT​CGA​AAC​AG-3 and reverse primer: 5-CTC​ACG​AGG​CCA​CTG​ATA​CCT​A-3; TNFα: forward primer:5-CATCTTCTCAAAATTCGAGTGACAA-3 and reverse primer:5-TGGGAGTAGAGGTACAACCC-3; IL-6: forward primer:5-GCTATGAAGTTCCTCTCCGC-3 and reverse primer: 5-CTA​GGT​TTG​CCG​AGT​AGA​TC-3; β-Actin: forward primer: 5-GAC​CTG​ACT​GAC​TAC​CTC-3 and reverse primer: 5-GCT​CAG​CGA​GGC​CAG​GAT​G-3.

### Conditioned medium

BV2 cells were pretreated with SA (5 μM) for 4 h, LPS (100 ng/mL) was added without changing the medium for 6 h, then original culture medium was abandoned and replaced with 1 mL of new culture medium for another 24 h. The culture medium was collected and centrifuged at 1,000 rpm for 5 min, the supernatant was added to corresponding SH-SY5Y cells of moderate density. After 24 h, the cells status were observed, cells were collected and cleaved-caspase three protein was detected in the extract.

### Molecular docking

Inflammatory pathway proteins were searched by consulting the literature and the target proteins were downloaded from the protein Three-dimensional Structure Database PDB (http://www1.rcsb.org/). The receptor protein was preprocessed by pymol software, and exported to PDBQT format. Ligand small molecules were downloaded from the Pubchem database and converted into mol2 format by OpenBabel software. AutoDock Tools software was used to process ligand small molecules, and PDBQT format was exported. Autoduck Vina software was used to simulate the interaction between ligand and receptor and conduct molecular docking simulation. Binding energy was calculated to predict affinity, pymol software was used to visualize the docking results.

### Data analysis

All data are expressed as the mean ± standard error (mean ± SD), and GraphPad Prism software was used for statistical analysis. Statistical significance was evaluated by t-tests for comparing two groups and one-way ANOVA for all groups. Differences were considered statistically significant at *p*-values of < 0.05.

## Results

### SA inhibited BV2 microglia activation induced by LPS

**FIGURE 1:**
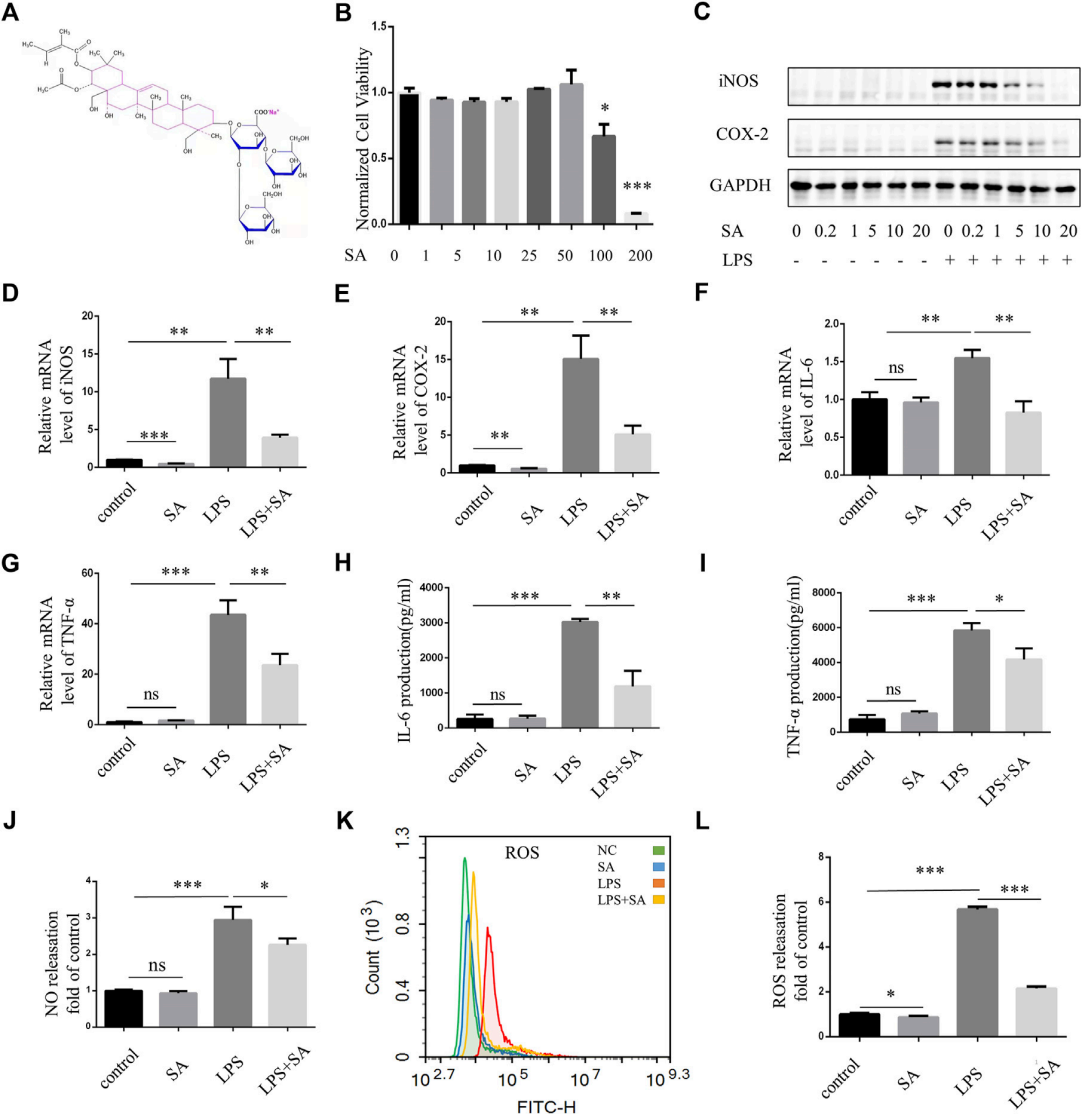
SA inhibited activation of BV2 microglia induced by LPS. **(A)** Chemical structural formula of sodium aescinate. **(B)** CCK8 assay was performed to examine the effect of SA to cell viability. BV2 cells were treated by different concentrations of SA (0–200 μM) for 24 h **(C)** BV2 cells were pretreated with different concentrations of SA (0–20 µM) for 12 h, then add LPS (100 ng/mL) for 12 h. Cell lysates were used to detect the protein levels of iNOS, COX2 and GAPDH by western blot. **(D–G)** BV2 cells were pretreated with SA (5 μM) for 18 h and treated with LPS (100 ng/mL) for another 6 h. Cell samples were collected, Then RNA was extracted to detect the levels of mRNA of iNOS, COX-2, TNF-α, and IL-6 by qRT-PCR. Date are presented as mean ± SD,**p < 0.01, ***p < 0.001, *n* = 3 per group. **(H–I)** BV2 cells were pretreated with SA for 12 h and treated with LPS for another 12 h. The levels of cytokines TNFα and IL-6 in the supernatant were measured by ELISA. Date are presented as mean ± SD, *p < 0.05, **p < 0.01, ***p < 0.001, *n* = 3 per group. **(J)** Nitrite concentration was measured by Griess assay, BV2 cells were pretreated with SA (5 μM) for 12 h, and then treated with LPS (100 ng/mL) for 12 h. The supernatant was taken and the corresponding Griess reagent was added, which was detected by a microplate reader. **(K)** After treatment with SA and LPS 12 h respectively, the cells were rinsed with D-Hank. The cells were incubated with DCFH-DA for 20 min at 37°C and detected by flow cytometry. **(L)** Statistical map of ROS release. Date are presented as mean ± SD, *p < 0.05, ***p < 0.001, *n* = 3 per group.

The chemical structural formula of SA was showed (Figure 1A). BV2 cells were immortalized mouse microglia cell lines. CCK-8 assay was used to detect the effect of different concentrations of SA on the viability of BV2 cells. When the concentration reached 50 μM, SA had no toxic effect on the viability of BV2 cells (Figure 1B). Pretreatment of SA dose-dependently inhibited LPS-induced expression of inflammatory proteins iNOS and COX-2 (Figure 1C). When the concentration of SA was 5 μM, SA had obvious anti-inflammatory effect and no cytotoxic to cell. Therefore, this concentration was used in the following experiments. qPCR experiment displayed that mRNA levels of iNOS, COX-2, TNF-α and IL-6 were increased by LPS stimulation. However, SA pretreatment could decrease the transcription of these genes (Figures 1D–G). ELISA (Enzyme-linked immunosorbent assay) showed that the release of TNF-α and IL-6 increased after LPS stimulation in BV2, while SA pretreatment suppressed their secretion (Figures 1H, I). NO destroys mitochondrial oxidative phosphorylation function which release ROS to exacerbate neuroinflammation and nerve tissue damage (Leng and Edison, 2021). SA inhibit LPS-induced NO release in BV2 (Figure 1J). Excessive ROS could cause lipid peroxidation, protein carbonylation, and DNA oxidation, lead to changes in membrane permeability and fluidity and membrane damage as well as ultimately apoptosis and tissue necrosis (Janbandhu et al., 2022). SA could inhibit LPS-induced ROS release in BV2 microglia (Figures 1K, L).

### SA inhibited NF-κB pathway activation of microglia

**FIGURE 2:**
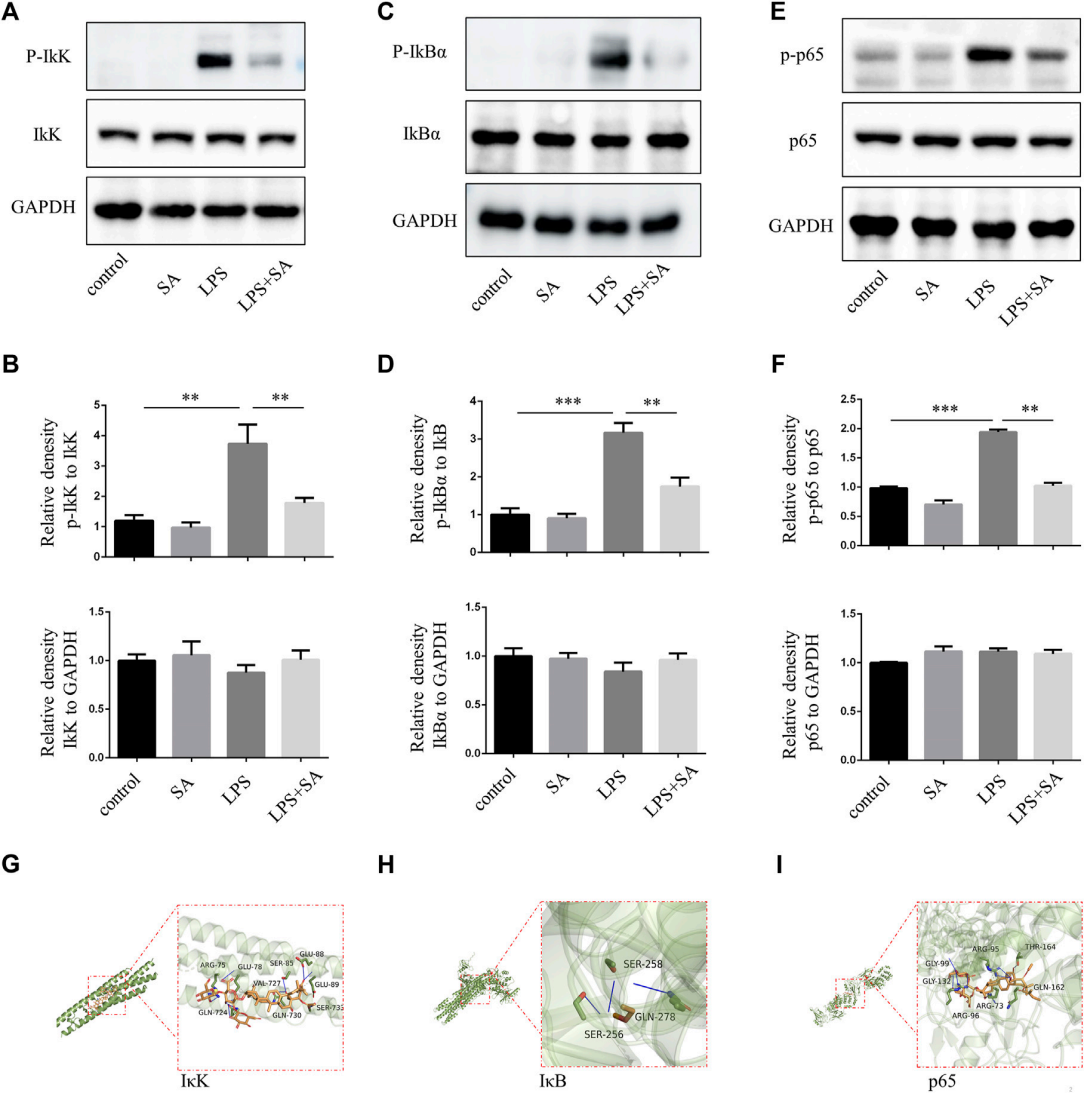
SA inhibited NF-κB activation of BV2 induced by LPS. **(A–F)** BV2 cells were pretreated with SA for 12 h. After 15 min with LPS (100 ng/mL), the proteins were extracted and the protein levels of IκK, p-IκK, IκB, p-IκB, p65, and p-p65 were measured by western blot assay. **(G–I)** Molecular docking of active compound SA and core genes IκK, IκB, p65. Date are presented as mean ± SD,**p < 0.01, ***p < 0.001, *n* = 3 per group.

In classical NF-κB pathway, IκK is activated after LPS stimulation, and subsequently phosphorylates of IκB and p65. In our study, SA reduced the phosphorylation of IκK, IκB and p65 in BV2 induced by LPS (Figures 2A–F). Molecular docking technique simulated the binding ability of SA to IκK, IκB and p65. The affinity was predicted by calculating the binding energy. The binding energy was lower than zero, which indicated that the small molecule ligand had spontaneous binding ability to the large molecule receptor. When the binding energy was lower than −5 kcal/mol, it indicated that the ligand had better binding ability to the receptor (Pinzi and Rastelli, 2019). In this study, the maximum binding capacity of SA to IκK, IκB and p65 was −6.8, −6.6, −8.2 kcal/mol, all of which showed strong affinity (Figures 2G–I). Both molecular docking techniques and experiments certificated that SA inhibited microglia activation through NF-κB signaling pathway.

### SA inhibited the activation of primary microglia *in vitro*

**FIGURE 3:**
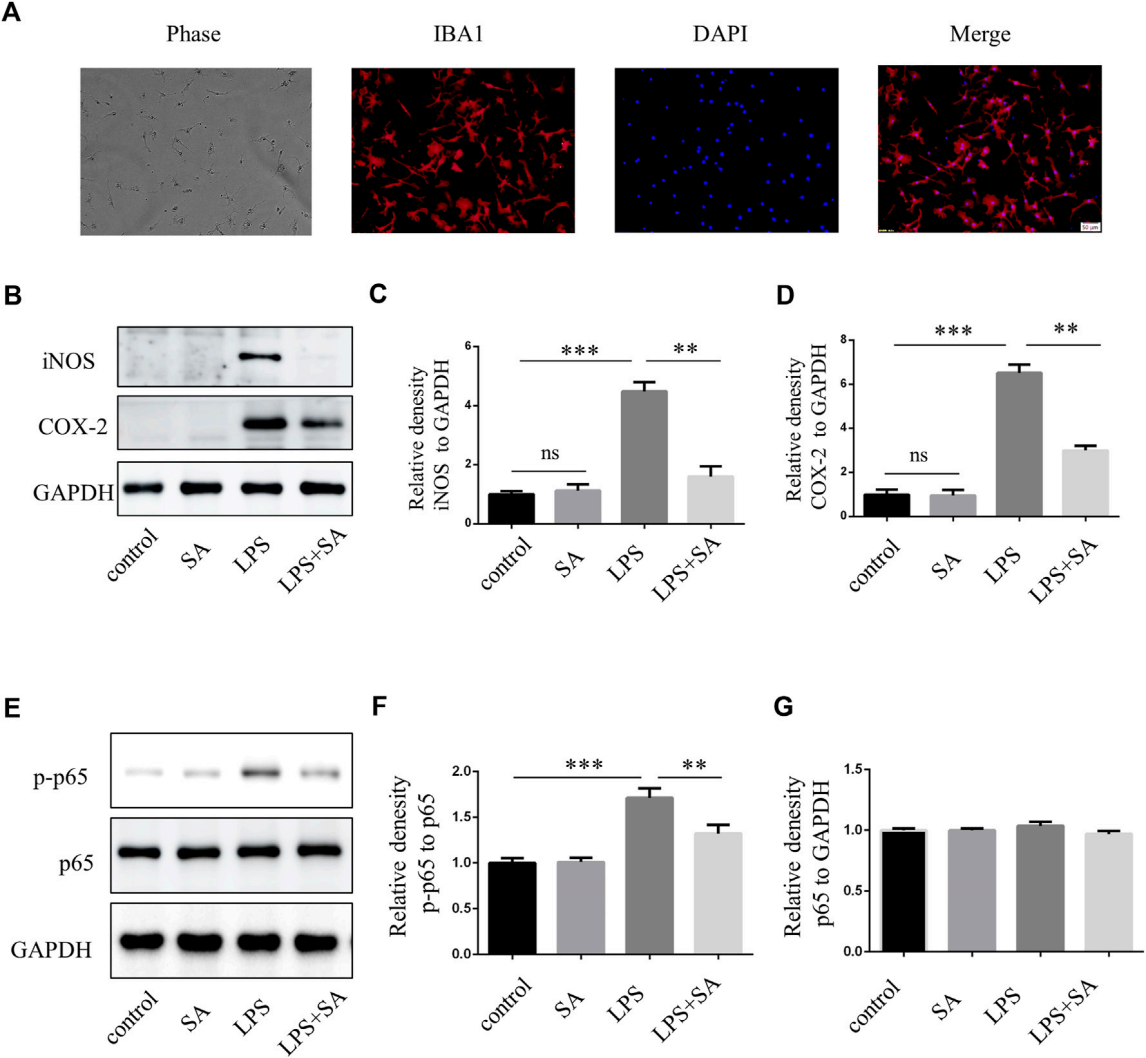
SA inhibited LPS-induced activation of primary microglia *in vitro*. **(A)** Primary microglia cells were stained with IBA1 antibody. **(B–D)** BV2 cells were pretreated with SA (5 µM) for 12 h and LPS (100 ng/mL) was added for another 12 h. Proteins were extracted and the protein levels of iNOS, COX-2 and GAPDH were measured by western blot assay. **(E–G)** Primary microglia were pretreated with SA for 12 h. After adding LPS (100 ng/mL) for 15 min, the protein was extracted and the protein levels of p65, p-p65 were detected by western blot assay. Date are presented as mean ± SD, **p < 0.01, ***p < 0.001, *n* = 3 per group. Scale bar, 50 µm.

BV2 cells were mouse microglia cells immortalized by retrovirus-mediated transfection of v-RAF/v-MYC, which were somewhat different from primary microglia in terms of morphology, proliferation ability, and response to stress (Yan et al., 2018; Prinz et al., 2019). Therefore, in order to increase confidence in experimental results, primary microglia were extracted from the cerebral cortex of neonatal mouse. The effect of SA was examined by inhibiting primary microglia-mediated inflammation. The purity of primary microglia was detected by microglia marker IBA1 (Figure 3A). Further, SA significantly reduced the expression of iNOS and COX-2 in primary microglia stimulated by LPS (Figures 3B–D). Similarly, p65 phosphorylation was enhanced after LPS treatment in primary microglia, while SA alleviated the phosphorylation of p65 (Figures 3E–G). All these results demonstrated that SA could inhibit the activation of primary microglia induced by LPS.

### SA inhibited microglia activation induced by LPS *in vivo*

**FIGURE 4:**
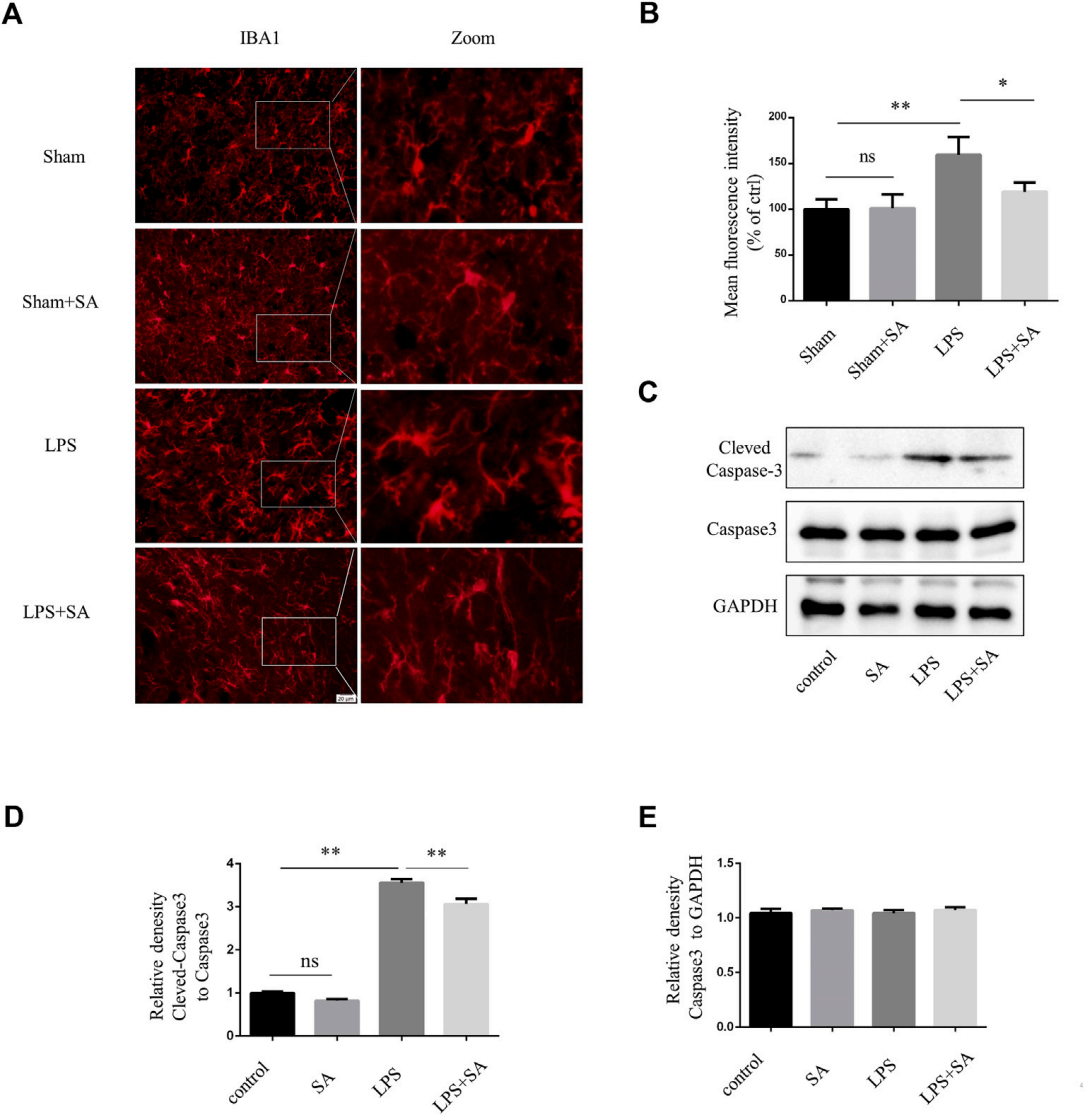
SA inhibited LPS-induced microglial activation *in vivo* and exerts neuroprotective effects. **(A)** Mice were stereotaxically injected with 2 μL LPS (1 mg/mL) into the ventricles, followed by SA (1 mg/kg) once a day for seven consecutive days. The brains were cut into brain slices and stained by antibody IBA1. **(B)** Statistical plot of IBA1 fluorescence intensity. **(C–E)** Caspase3 and Cleved-caspase3 were extracted from SH-SY5Y cells and detected by western blot assay. Date are presented as mean ± SD,*p < 0.05, **p < 0.01, *n* = 3 per group. Scale bar, 20 µm. Coronal slices.

A neuroinflammatory model is established by stereotaxic injection of LPS into the ventricles of mouse (Guo et al., 2019). Microglia were significantly activated after LPS injection. However, microglia activation were attenuated after SA administration (Figures 4A, B). Active caspase-3 in cells was an important apoptosis indicator (Nagata, 2018). LPS treatment of BV2 conditioned medium could induce caspase-3 activation of SH-SY5Y cell. SA + LPS pretreatment of BV2 conditioned medium had a low activation level of caspase-3 (Figures 4D, E). The above results indicated that SA could exert a neuroprotective effect by inhibiting the activation of microglia induced by LPS.

### SA inhibited microglia activation in TBI model

**FIGURE 5:**
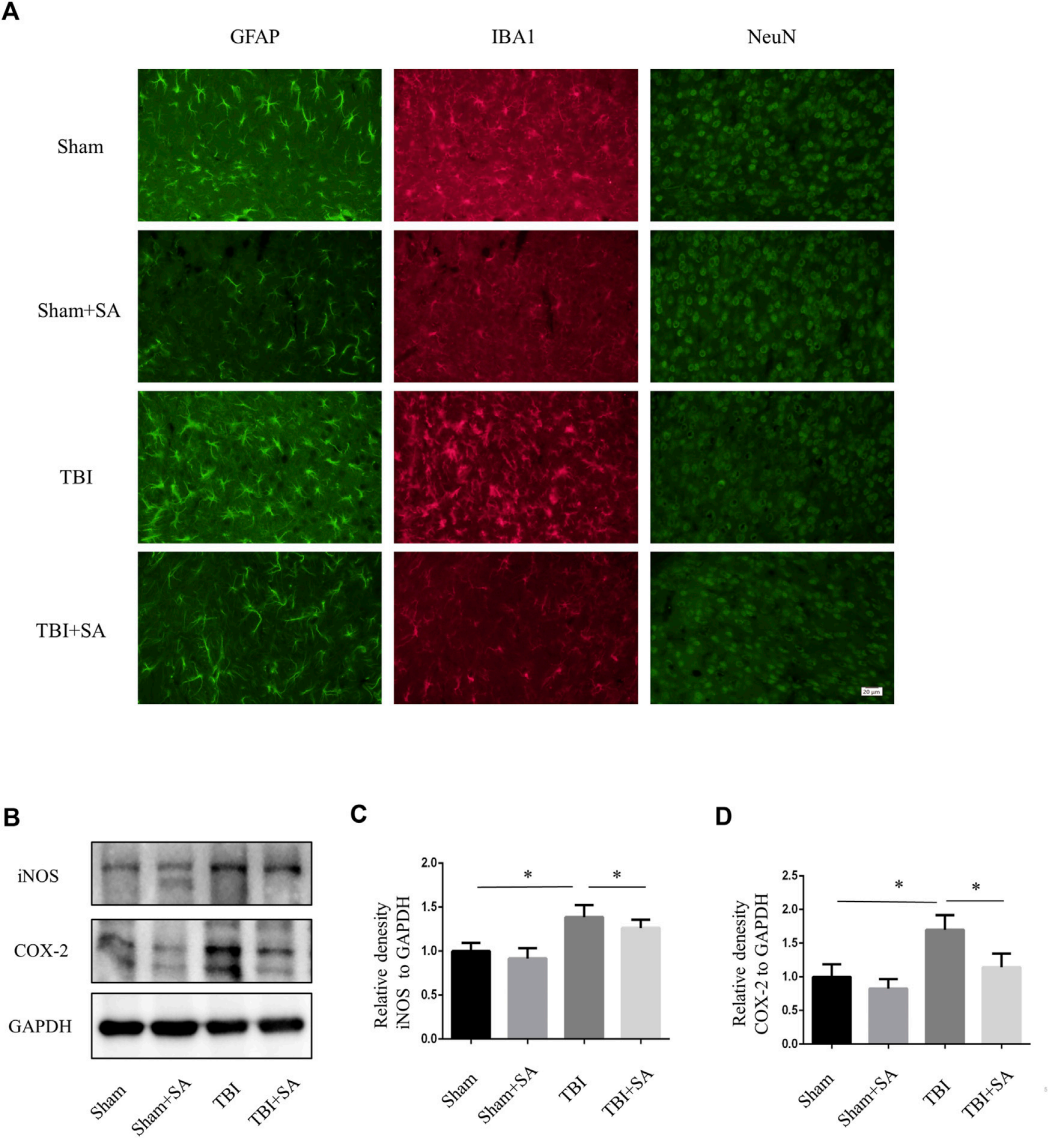
SA inhibits activation of glial and expression of inflammatory proteins in cortical tissue TBI mice. **(A)** Mice were subjected to TBI and received SA (1 mg/kg) and vehicle once a day for seven consecutive days. The brains were cut into brain slices and stained by antibody GFAP, IBA1 and NeuN. **(B)** Cortical tissues were taken from TBI model mouse animals, the protein levels of iNOS, COX-2 and GAPDH were detected using western blot. **(C–D)** Statistical plots of iNOS and COX-2 inflammatory protein expression. Date are presented as mean ± SD, *p < 0.05, **p < 0.01, *n* = 3 per group. Scale bar, 20 µm. Coronal slices.

**FIGURE 6:**
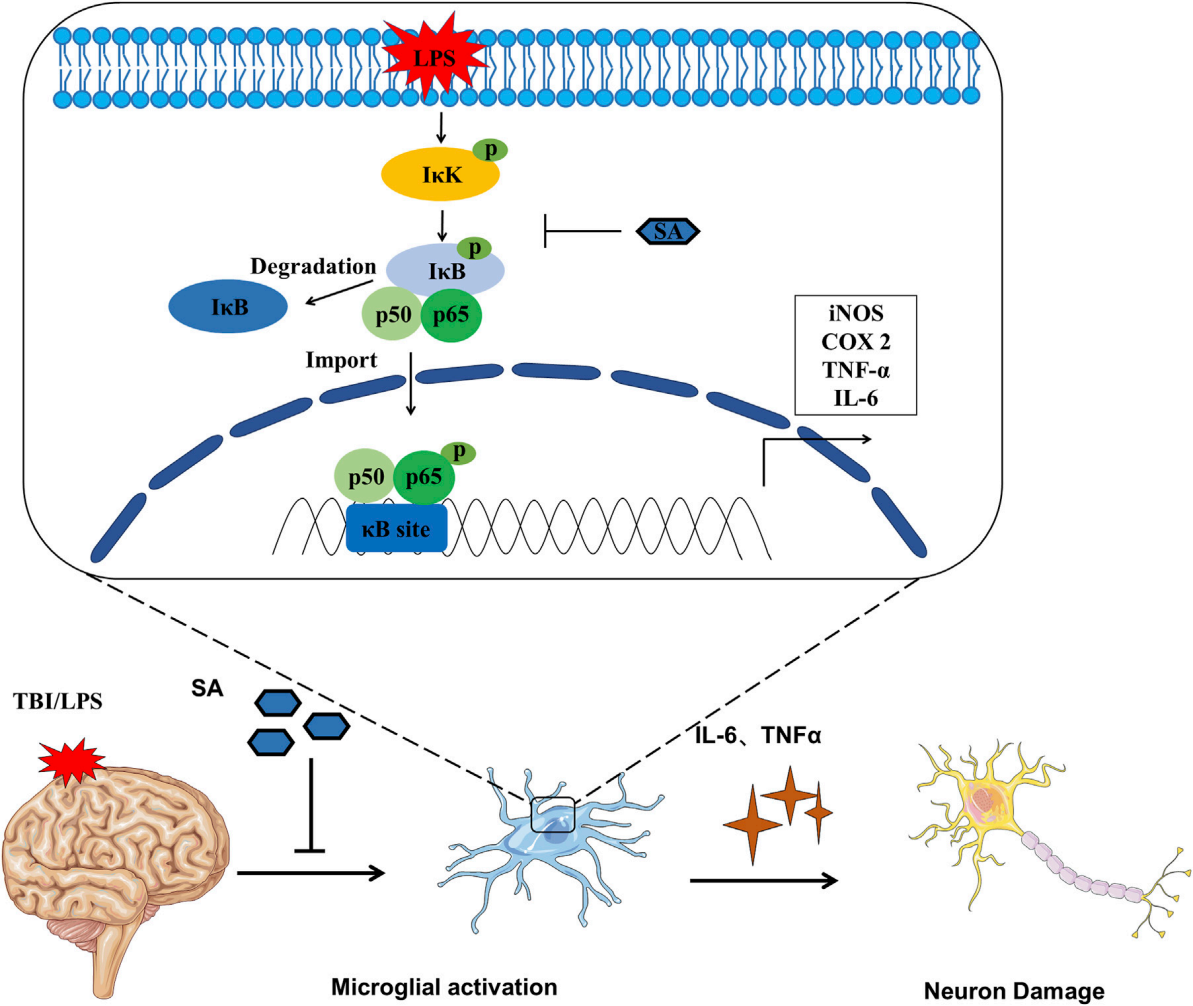
Schematic diagram SA inhibiting microglia activation and exerting neuroprotective effects.

Activation of microglia and damage of neuron could also be observed in TBI mouse cortex (Witcher et al., 2021). SA inhibited microglia activation and neuron damage (Figure 5A). Next, Cortical tissues were extracted to perform protein quantification. The expression of inflammatory proteins iNOS and COX-2 increased after TBI. However, the expression of inflammatory proteins was inhibited after SA administration. (Figures 6B–D). All the founds indicated that SA could inhibit the activation of microglia and protect neuron in TBI.

## Discussion

Microglia are resident immune cells of the central nervous system that sense environmental changes and maintain central nervous system homeostasis. During injury or disease, microglia cells are activated, at least in part, by signals from damaged neurons. Therefore, activated microglia contribute to neuroprotection and neuroinflammation. However, sustained microglial activation trigger a chronic neuroinflammatory response that disrupts neuronal health and disrupts communication between neurons and microglia (Haidar et al., 2022). Therefore, inhibition of microglial activation is a promising therapeutic approach for neuroinflammation.

Aescin is an active component extracted from the seeds of sauvignon mongolica, which has the effects of anti-exudation, anti-oxidation, and neuroprotection. Recently, SA was reported that it exerted neuroprotective effects in multiple models of neurological disease. SA provides neuroprotection in TBI *via* the Nrf2-ARE pathway (Zhang et al., 2020). SA reduces oxidative stress and provides neuroprotection in experimental traumatic spinal cord injury (Cheng et al., 2016). SA reduces ischemia-reperfusion (I/R) injury (Yang et al., 2011). SA increases the expression of apoptosis-related protein Bcl-2 and decrease the expression of Bax in cerebral ischemia-reperfusion injury (Fan et al., 2005). Although many reports have shown that SA has a neuroprotective effect, it is not clear whether it is related to microglia. Our study is to study whether SA exerts neuroprotective effects by inhibiting the activation of microglia.

In our study, SA inhibited microglia activation induced by LPS *in vivo* and vitro (Figure 1C; 3B; 4A). SA also inhibited microglia activation in TBI model (Figure 5). Microglia can be labeled by many proteins, such as IBA1, TMEM119, CX3CR1, F4/80, CD40, CD68 and CD11b/CD45. Ionized calcium-binding adapter molecule 1 (IBA1) involve in cell membrane folding and phagocytosis of activated microglia cells, which are often used to label microglia (Lier et al., 2021). Activated microglia have two phenotypes. M1 type is a cytotoxic phenotype with pro-inflammatory effect and phagocytic ability, induced by trauma, pathogenic microorganisms, LPS, IL-4 and other factors, can release iNOS, COX-2, TNF-α, IL-1β, IL-6 and other inflammatory mediators and inflammatory substances. M2 type can secrete anti-inflammatory factors and upregulate neuroprotective factors, promote cell proliferation and immune repair, and have neuroprotective effects (Dang et al., 2016).

As a marker of M1-type microglia, iNOS was necessary in activated microglia-mediated damage. When stimulated by LPS, microglia produce the inflammatory protein iNOS, which catalyze the production of L-citrulline and NO from L-arginine. NO is a neurotransmitter in the central and peripheral nervous system that played a beneficial role by inducing vasodilation, however, it exacerbated neuroinflammation and nerve tissue damage in the later stage (Cymerys et al., 2019). NO also causes metabolic hypoxia in the body and destroys mitochondrial oxidative phosphorylation function which release a large number of ROS to cause DNA damage (Tejero et al., 2019). It is reported that not only microglia but also astrocytes and neurons are able to express iNOS (Kozlov et al., 2017). We only proved that SA can inhibit the expression of iNOS in microglia cells, while it is not clear whether SA can inhibit the expression of iNOS in other cells in the brain of TBI mice. COX-2 was induced by external stimuli and catalyzed arachidonic acid to produce prostaglandins which participated in inflammatory responses. Pretreatment of SA inhibited LPS-induced expression of pro-inflammatory cytokines (IL-6, TNF-α), inducible enzymes (iNOS, COX-2), NO and ROS (Figures 1C–L).

In the classical NF-κB pathway, IκB bind with p50/p65 heterodimers in the cytoplasm and mask their DNA-binding domain (Barnabei et al., 2021). The activity of IκB is controlled by the phosphorylation of upstream IκB kinase (IκK). IκK phosphorylates IκBα, then IκBα is ubiquitinated and degraded, relieving the inhibition of p50/p65. Subsequently, p65 transfers into the nucleus, bind to κB sites, and activates downstream gene transcription (Yu et al., 2020). The phosphorylation levels of IκK, IκB, and p65 protein were significantly increased after LPS stimulation. However, the phosphorylation levels of IκK, IκB, and p65 protein were decreased after SA treatment (Figures 2A–F, 3E–G). Molecular docking technique was used to detect the complex interaction between SA and NF-κB signaling pathway protein targets. It was found that the binding energies of SA to IκK, IκB, and p65 were less than −5 kcal/mol, which showed a good binding ability. The bindings of SA to IκK, IκB, and p65 may inhibit their phosphorylation activation. These results confirmed that SA inhibited microglia activation through blocking the NF-κB pathway (Figures 2, 3E–G).

In this study, we also tested the role of SA in neuroprotection. SA pretreatment protected neuron *in vivo* and vitro (Figure 4C; Figure 5A). In this study, SA inhibited the excessive activation of microglia through NF-κB pathway, reduced the release of inflammatory mediators, and played a neuroprotective role. This study provides a new theoretical and experimental basis for improving the function of SA in the clinical treatment of neuroinflammation.

## Conclusion

In conclusion, the present study has revealed that SA treatment of microglia cells inhibited LPS induced NO and ROS production as well as iNOS and COX-2 mRNA and protein expression. SA also inhibited the production of pro-inflammatory cytokines, such as TNF-α and IL-6. SA inhibited TBI induced activation of mouse cortical microglia. These effects were exerted by attenuation of phosphorylation of NF-κB. This suggests that SA may exert neuroprotective effect by inhibiting microglia activation.

## Data Availability

The original contributions presented in the study are included in the article/Supplementary Material, further inquiries can be directed to the corresponding authors.

## Abbreviations

TBI; Traumatic brain injury, SA; Sodium Aescinate, ANOVA; Analysis of variance, DMEM; Dulbecco’s modified Eagle medium, DF12; Dulbecco’s Modified Eagle Medium/Nutrient Mixture F-12, DMSO; Dimethyl sulfoxide, LPS; Lipopolysaccharide, PBS, Phosphate-buffered saline, TNF; Tumor necrosis factor, IL-6; Interleukin-6, iNOS; Nitric Oxide Synthase, COX-2; Cyclooxygenase-2, NO; nitric oxide, ROS; reactive oxygen species, PDL; Poly-D-lysine, NF-κB; nuclear factor-kappa B, PFA; Paraformaldehyde, TG; Triacylglycerol, PG; prostaglandin, WB; Western blot, SDS-PAGE; Sodium Dodecyl Sulfate-Polyacrylamide gel electrophoresis, ELISA; Enzyme-linked immunosorbent assay.
