# Supplementary figures and images for: Sodium aescinate inhibits microglia activation through NF-κB pathway and exerts neuroprotective effect
Source: Front Pharmacol. 2023 Jan 26;14:1086429. doi: 10.3389/fphar.2023.1086429 (PMC9908748; doi:10.3389/fphar.2023.1086429)

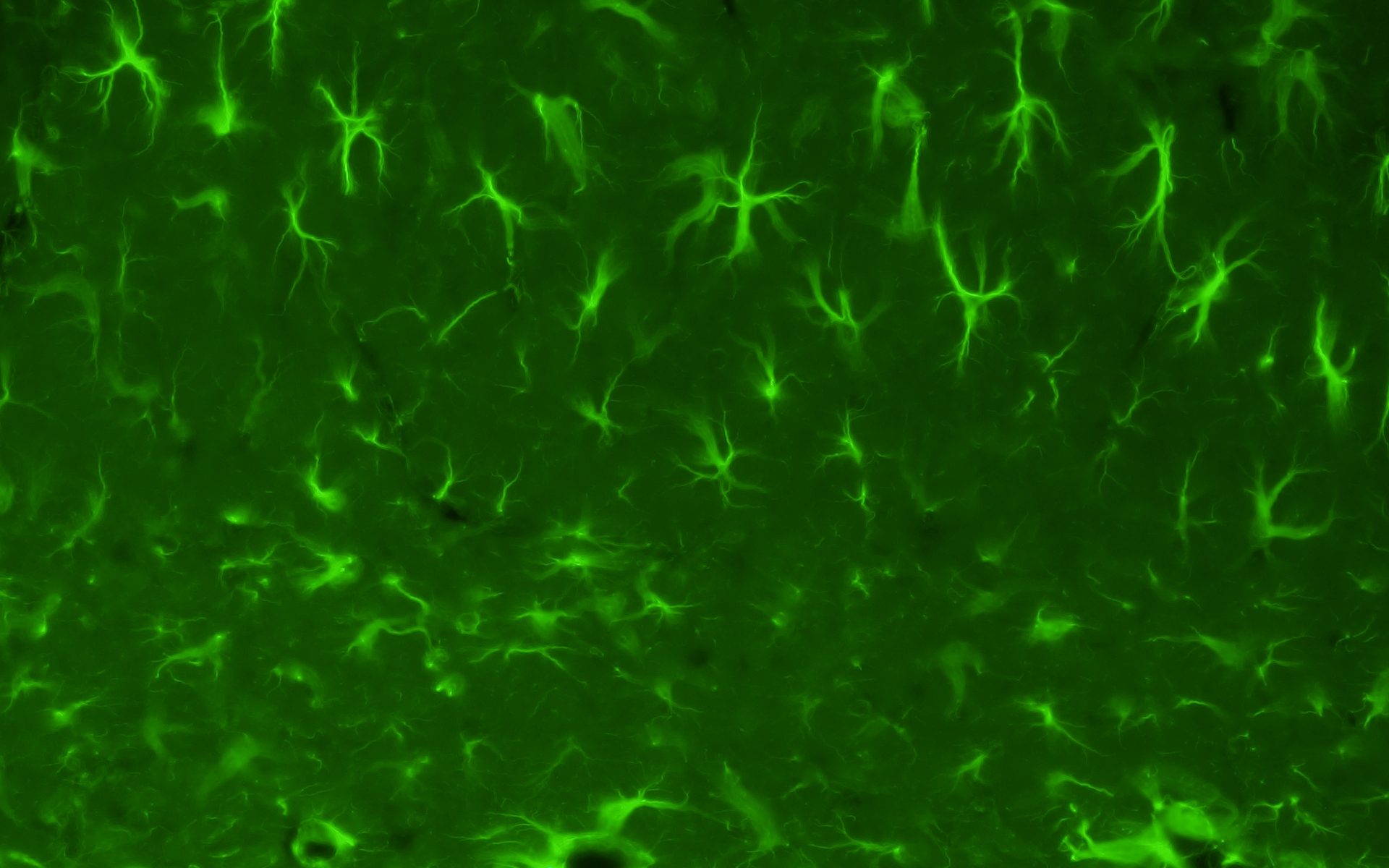

Supplement: Supplementary file 2 [file DataSheet3.ZIP › Figure 5A/Sham GFAP .jpg]

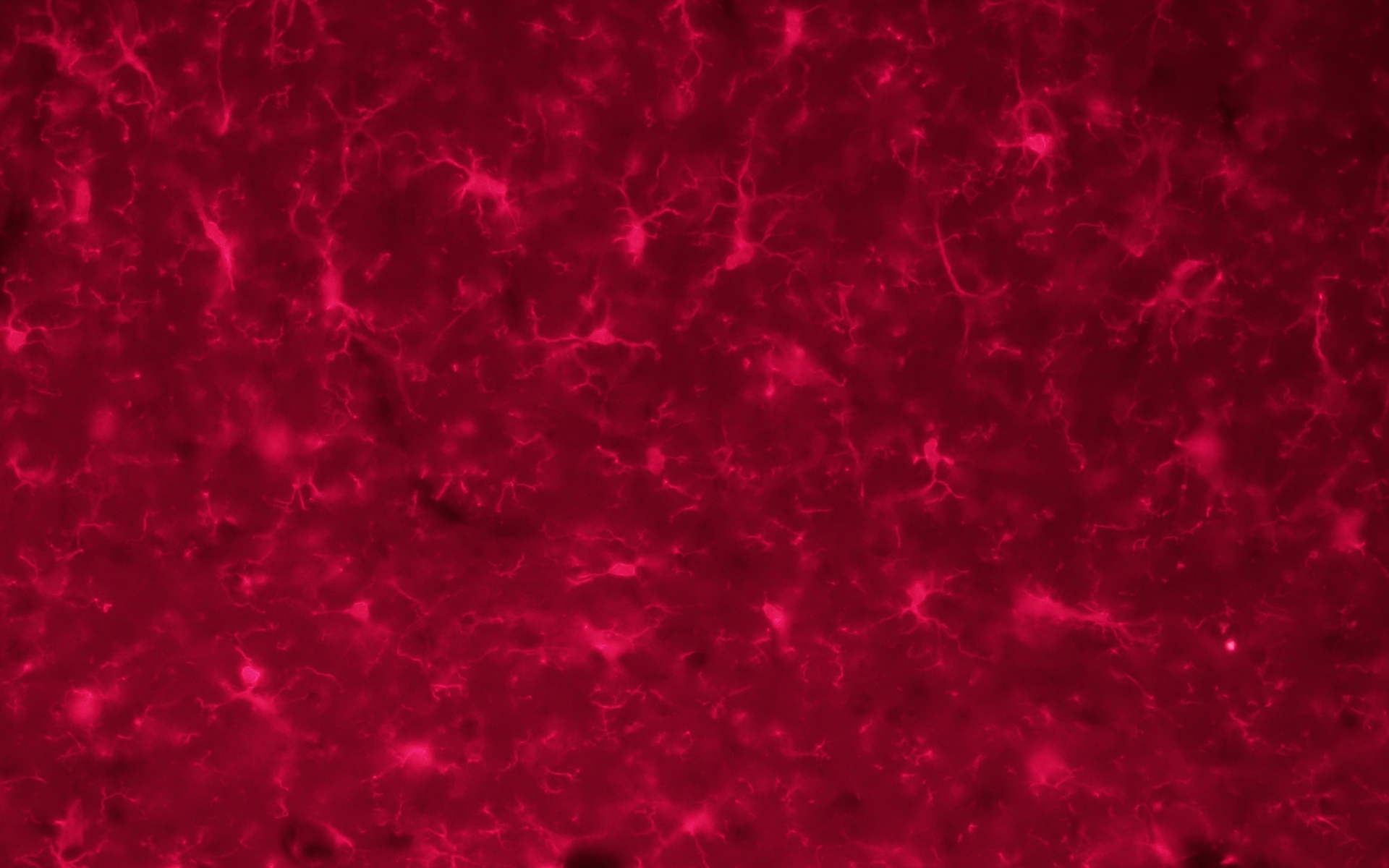

Supplement: Supplementary file 2 [file DataSheet3.ZIP › Figure 5A/Sham IBA1.jpg]

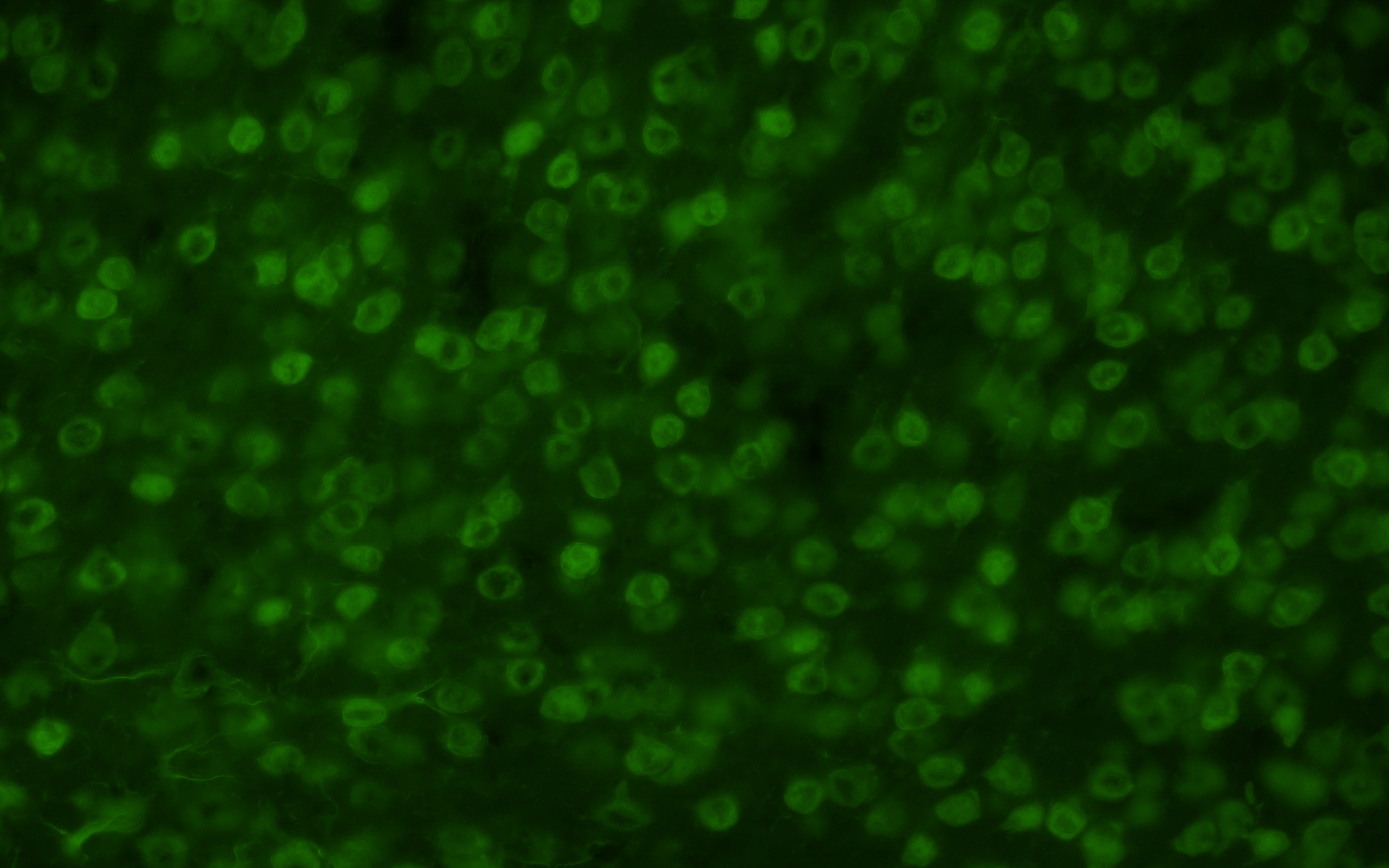

Supplement: Supplementary file 2 [file DataSheet3.ZIP › Figure 5A/Sham NeuN.jpg]

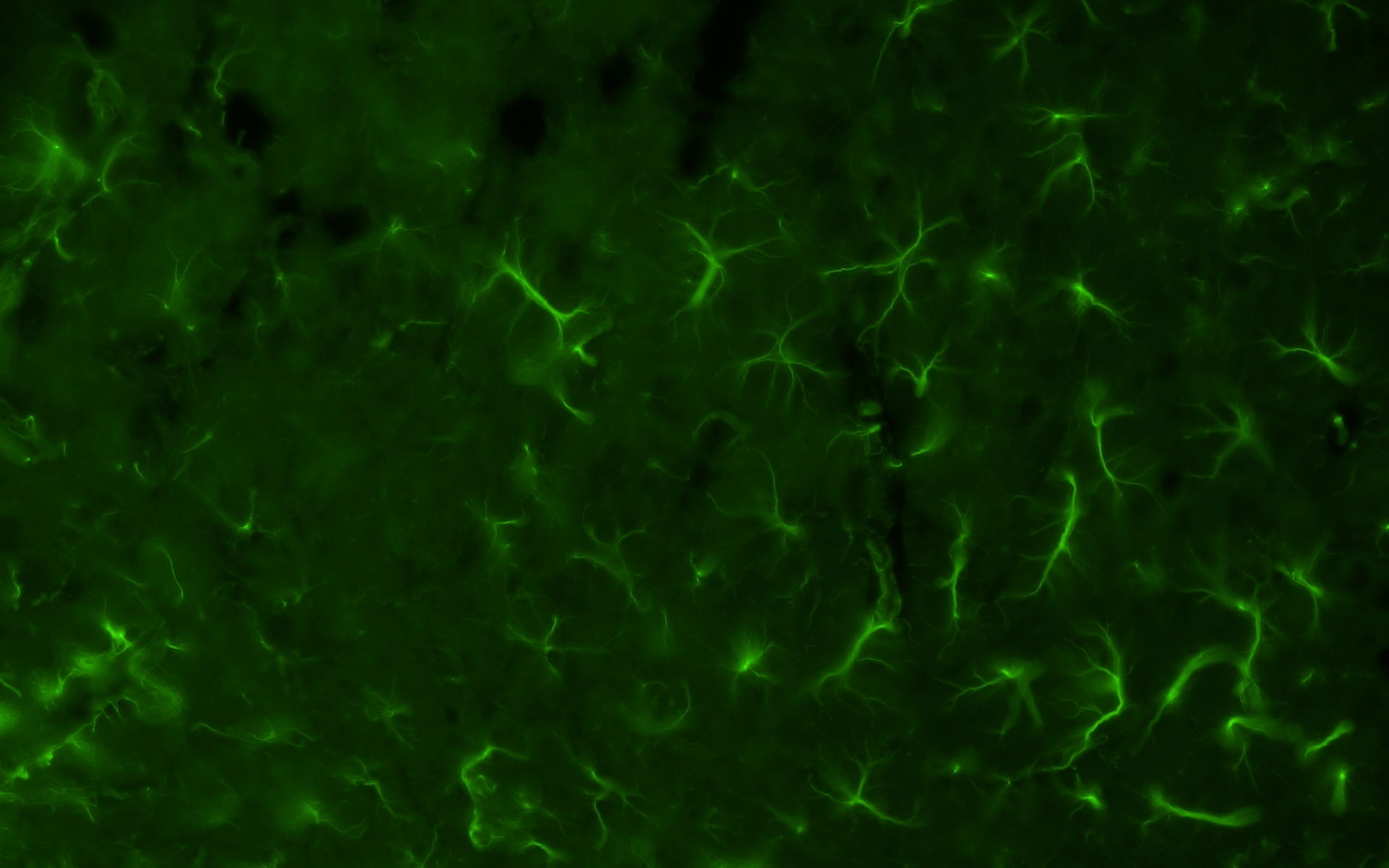

Supplement: Supplementary file 2 [file DataSheet3.ZIP › Figure 5A/Sham SA GFAP.jpg]

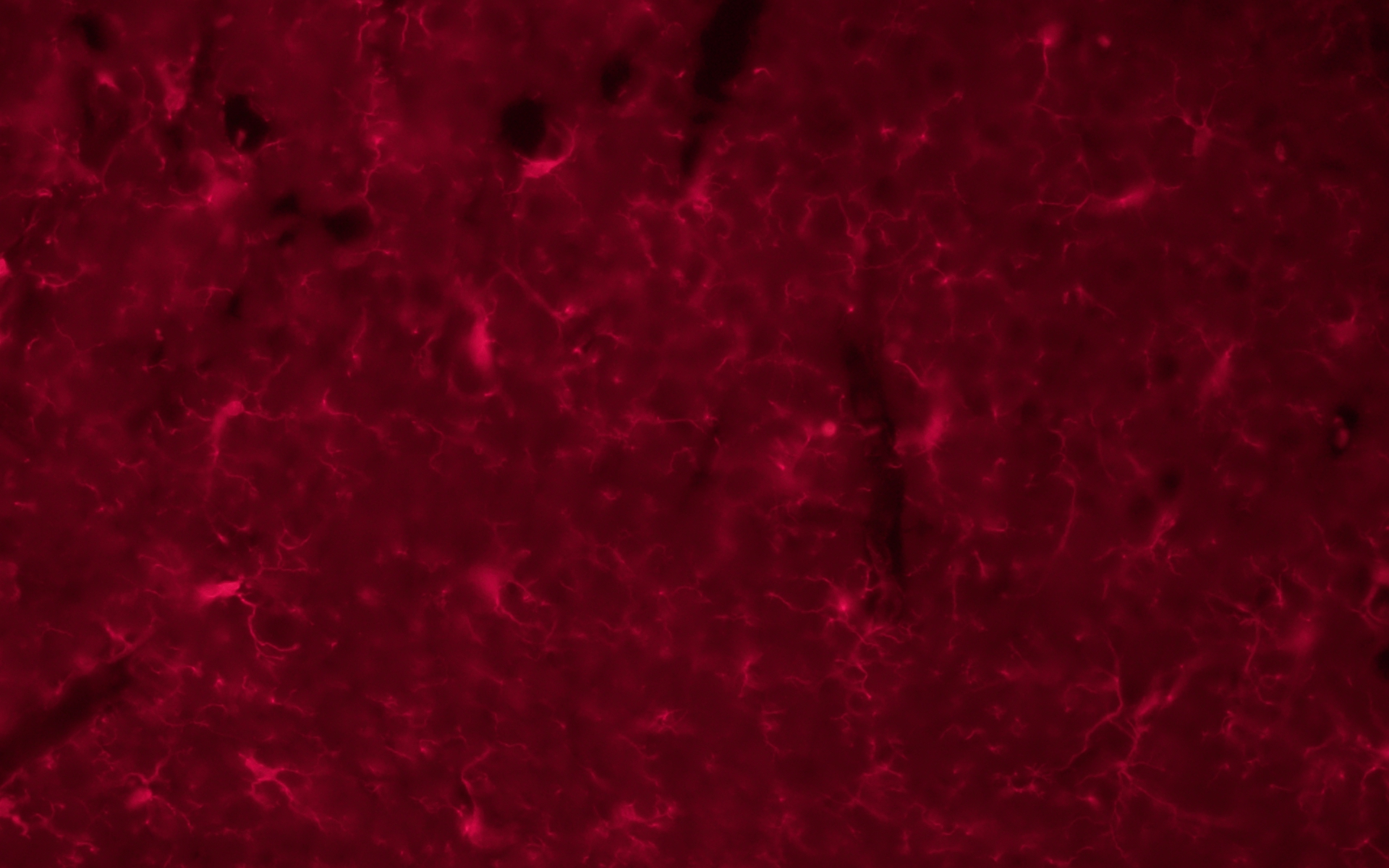

Supplement: Supplementary file 2 [file DataSheet3.ZIP › Figure 5A/Sham SA IBA1.jpg]

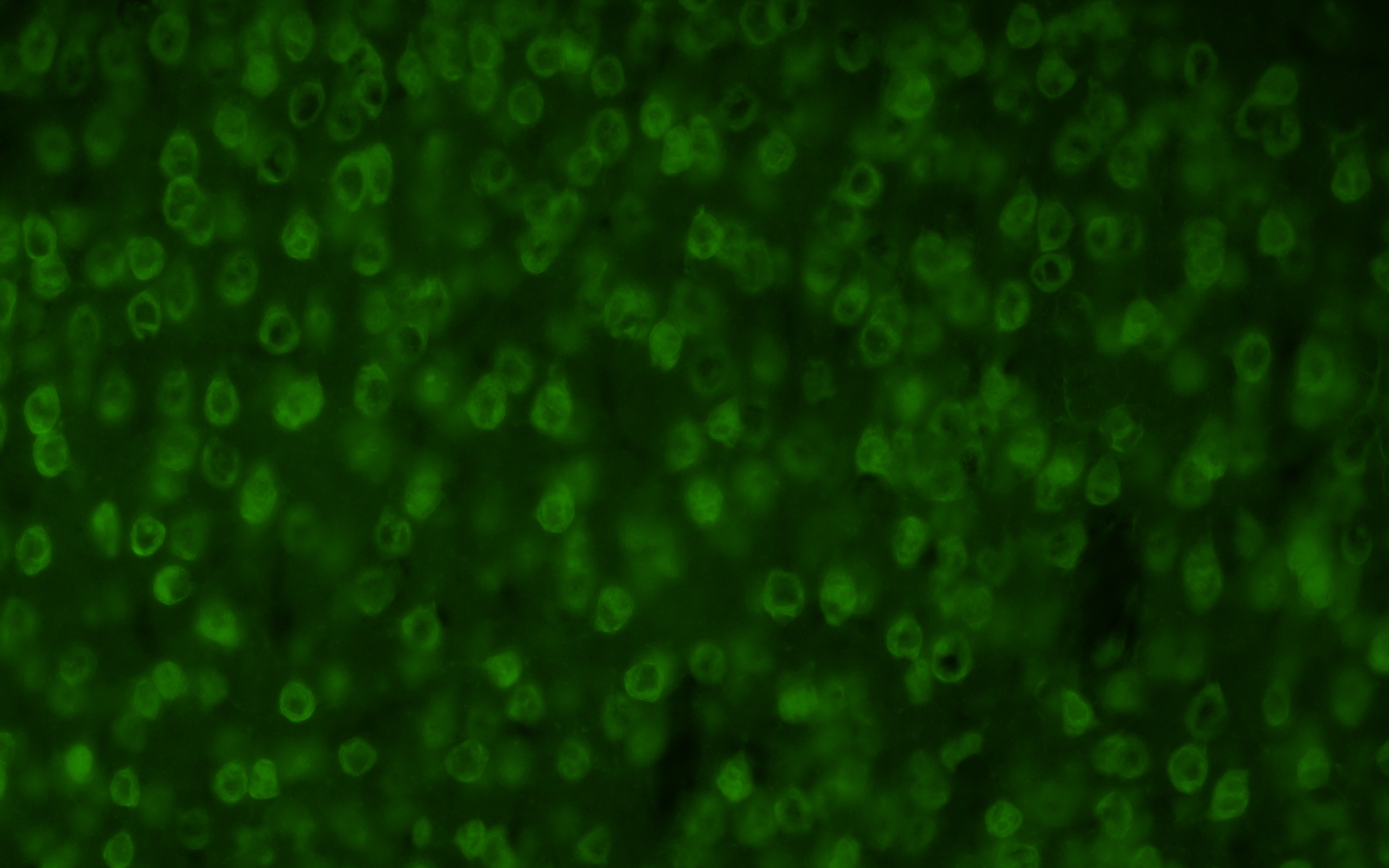

Supplement: Supplementary file 2 [file DataSheet3.ZIP › Figure 5A/Sham SA NeuN.jpg]

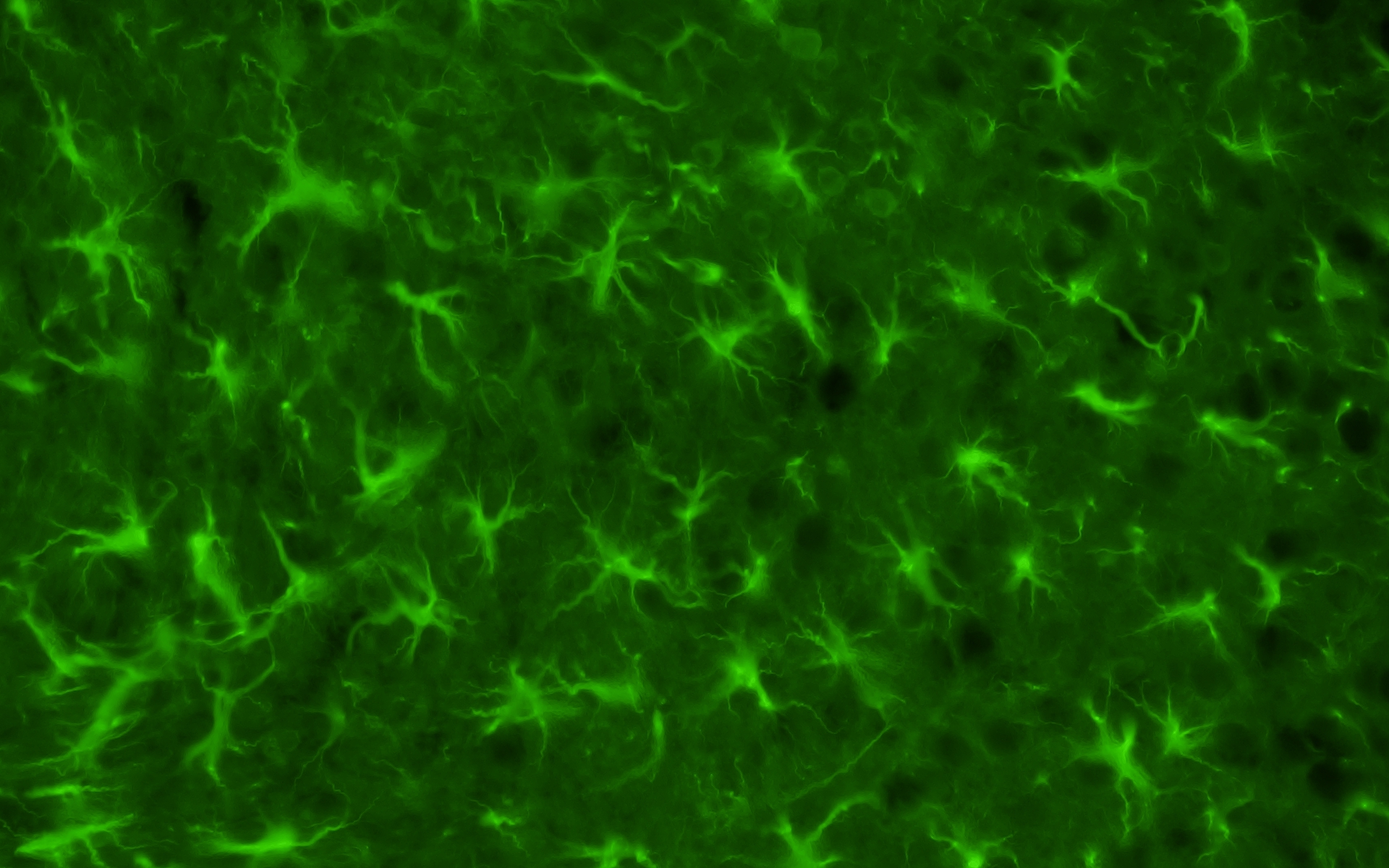

Supplement: Supplementary file 2 [file DataSheet3.ZIP › Figure 5A/TBI GFAP.jpg]

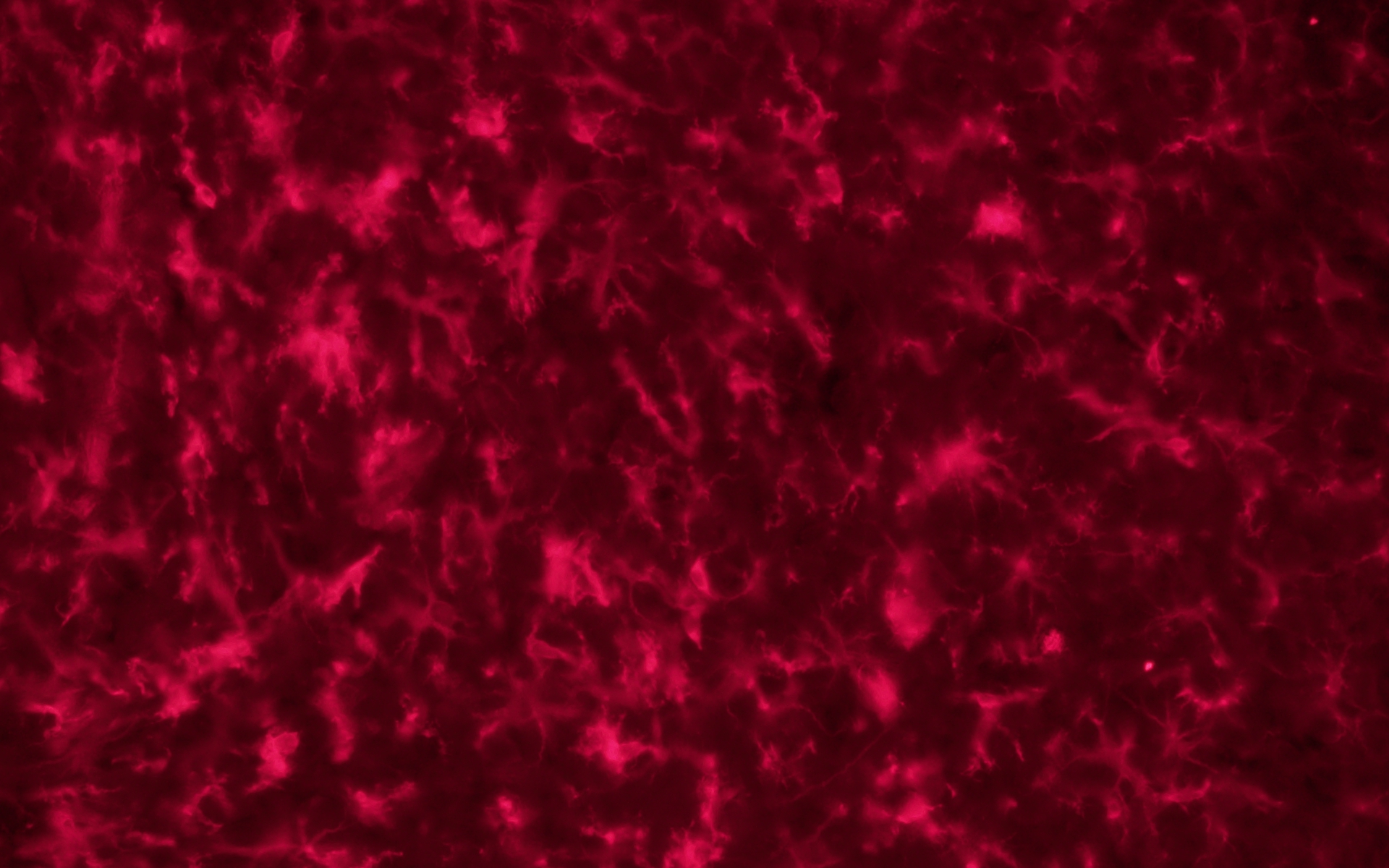

Supplement: Supplementary file 2 [file DataSheet3.ZIP › Figure 5A/TBI IBA1.jpg]

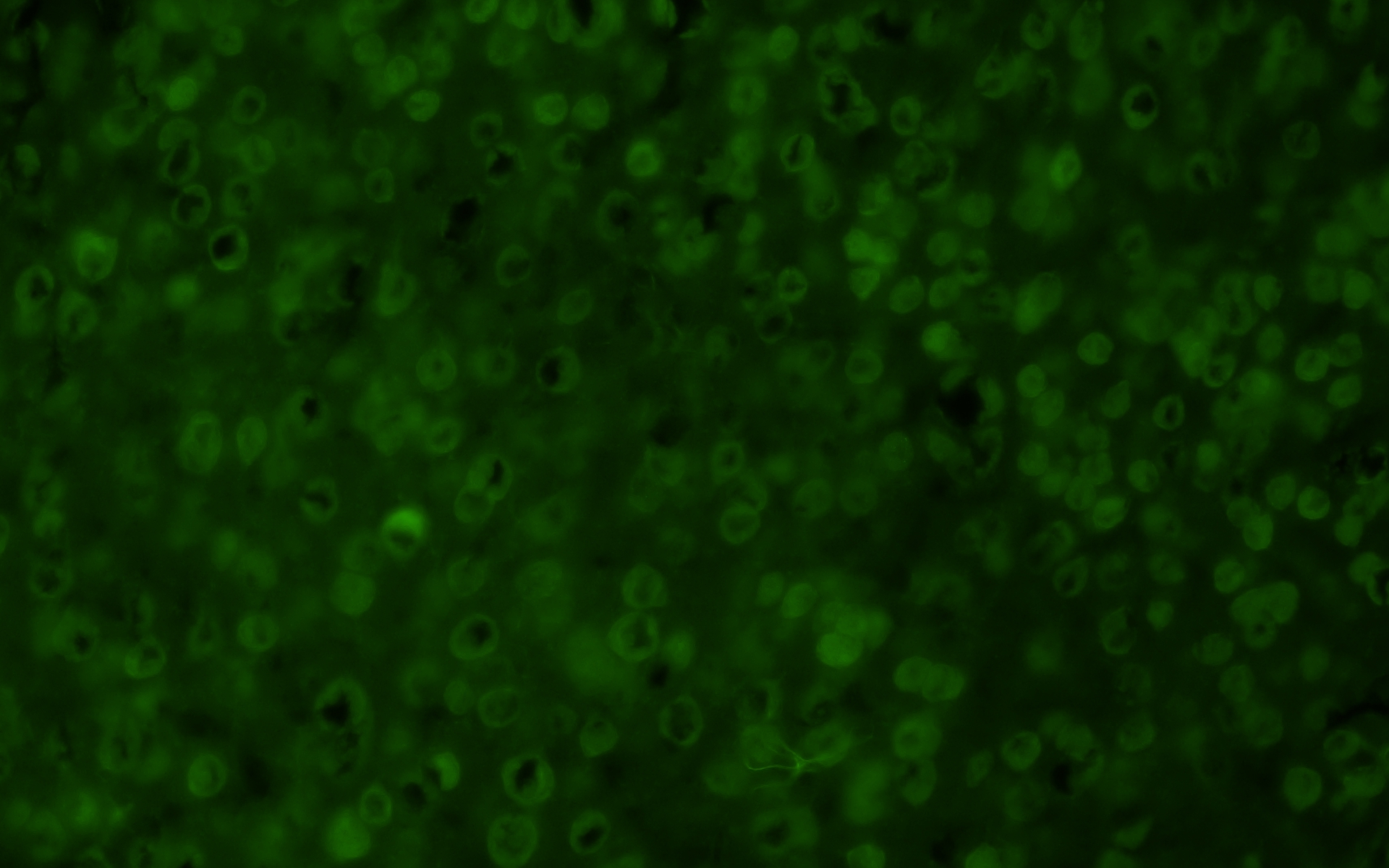

Supplement: Supplementary file 2 [file DataSheet3.ZIP › Figure 5A/TBI NeuN.jpg]

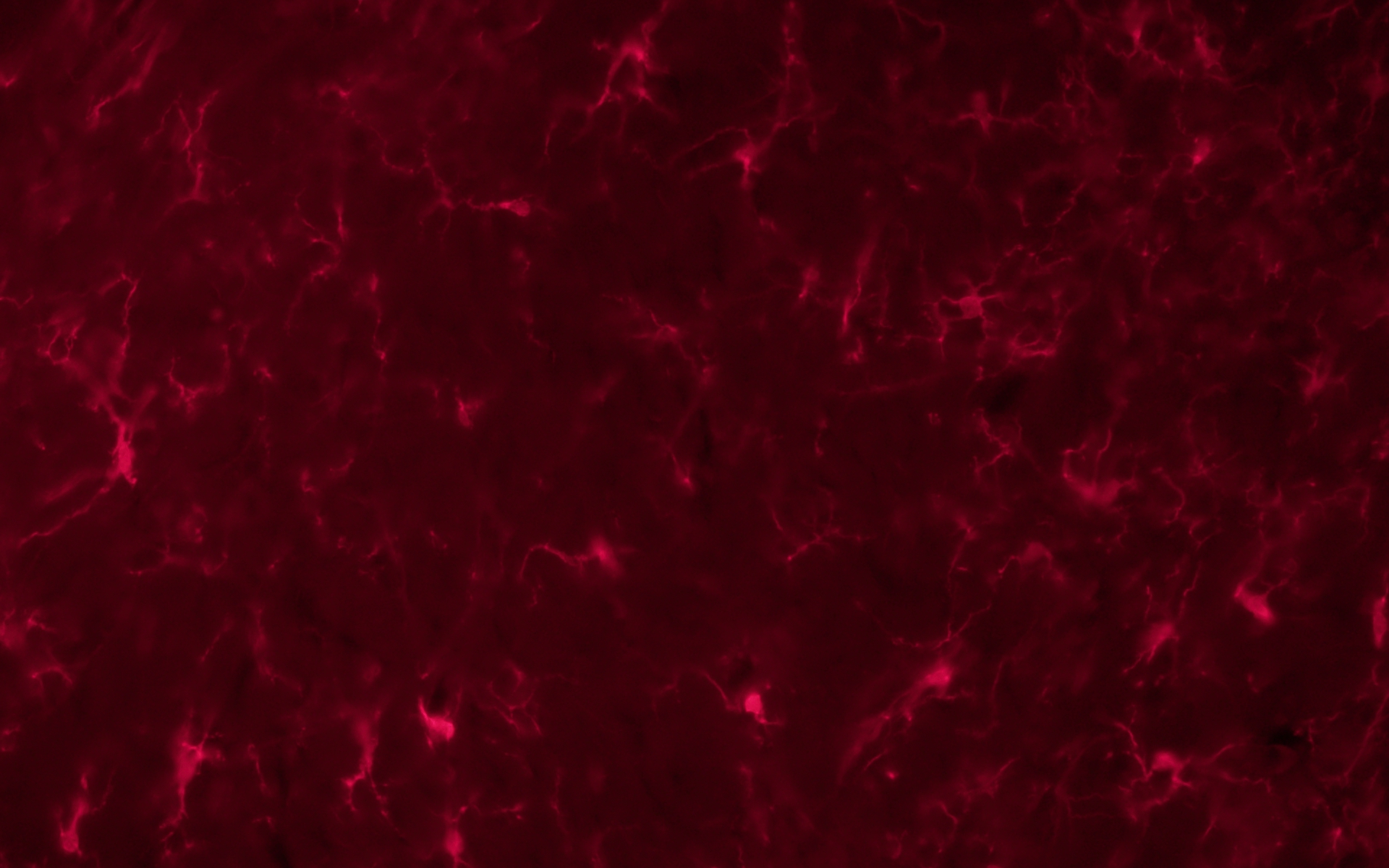

Supplement: Supplementary file 2 [file DataSheet3.ZIP › Figure 5A/TBI SA IBA1.jpg]

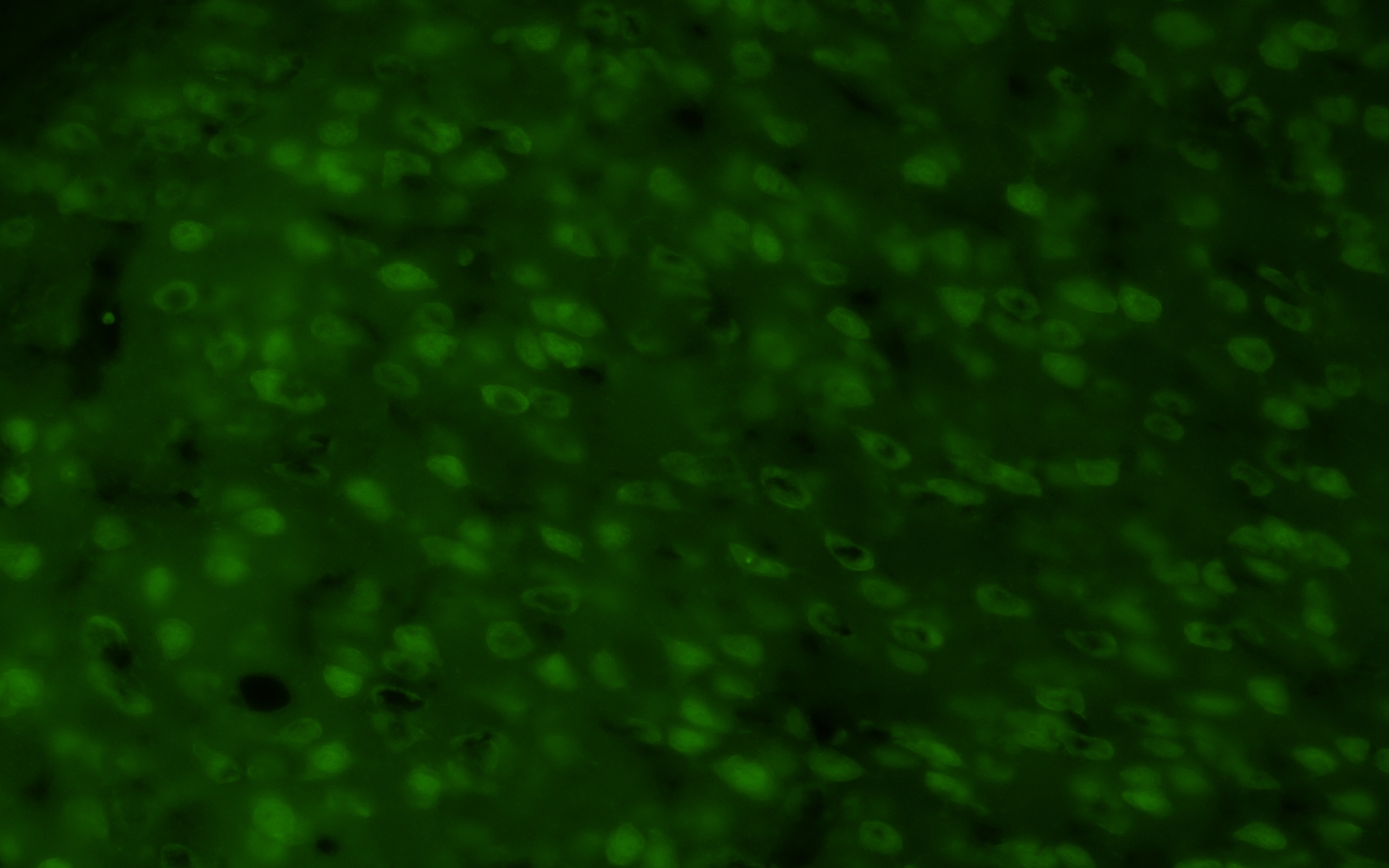

Supplement: Supplementary file 2 [file DataSheet3.ZIP › Figure 5A/TBI SA NeuN.jpg]

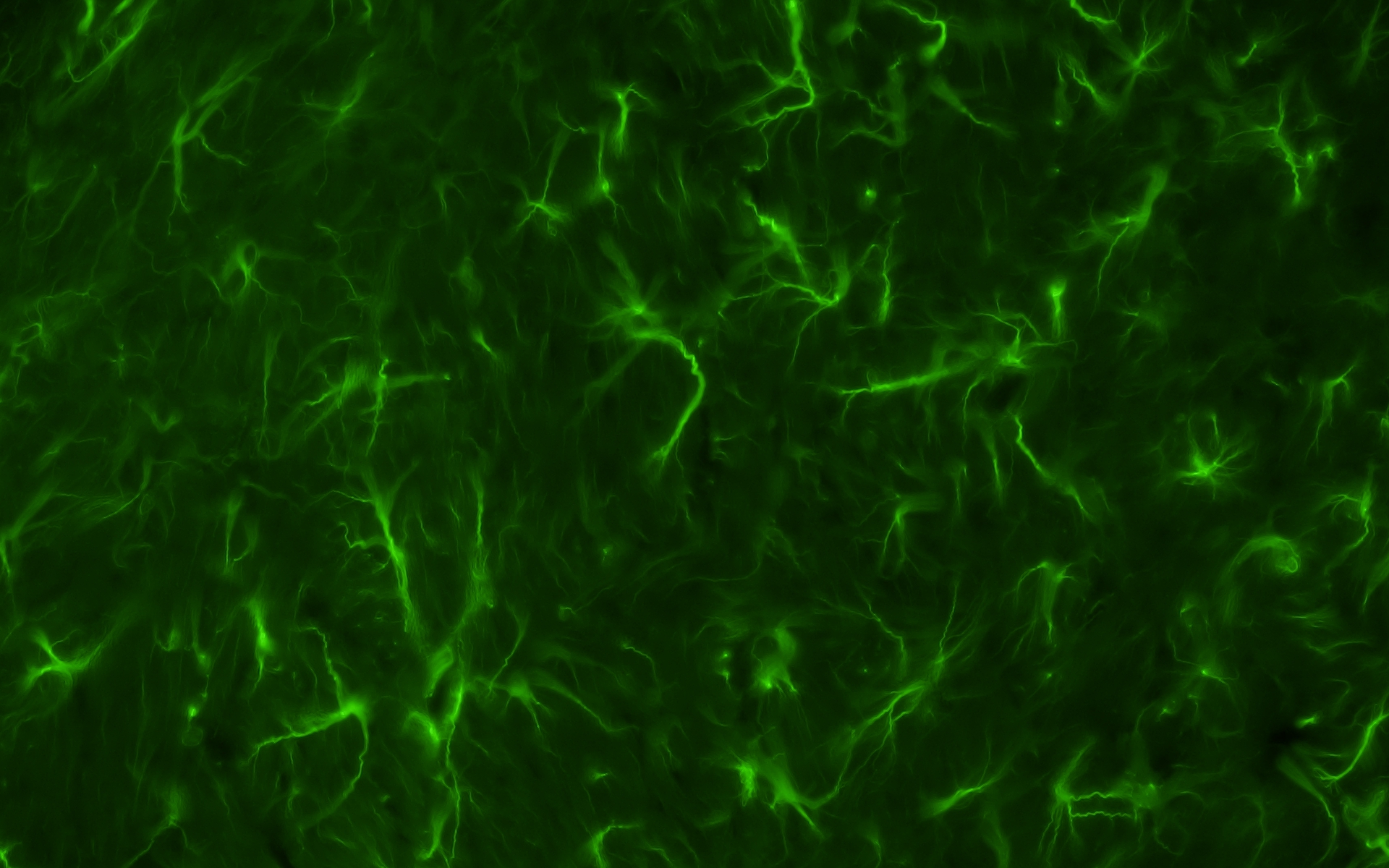

Supplement: Supplementary file 2 [file DataSheet3.ZIP › Figure 5A/TBI SA GFAP.jpg]

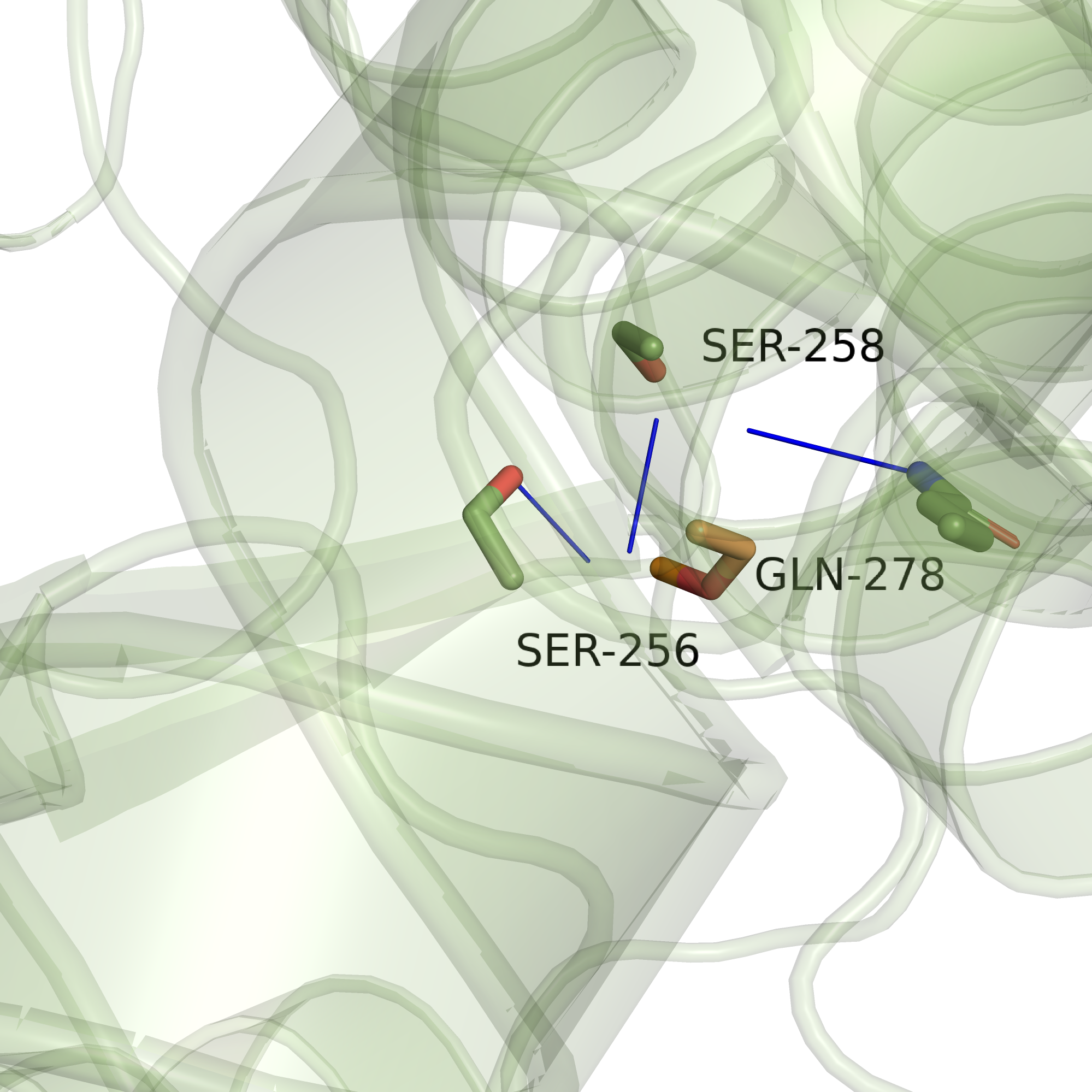

Supplement: Supplementary file 5 [file DataSheet4.ZIP › Figure2 G-I Molecular docking/IkB/ikb/dock1.png]

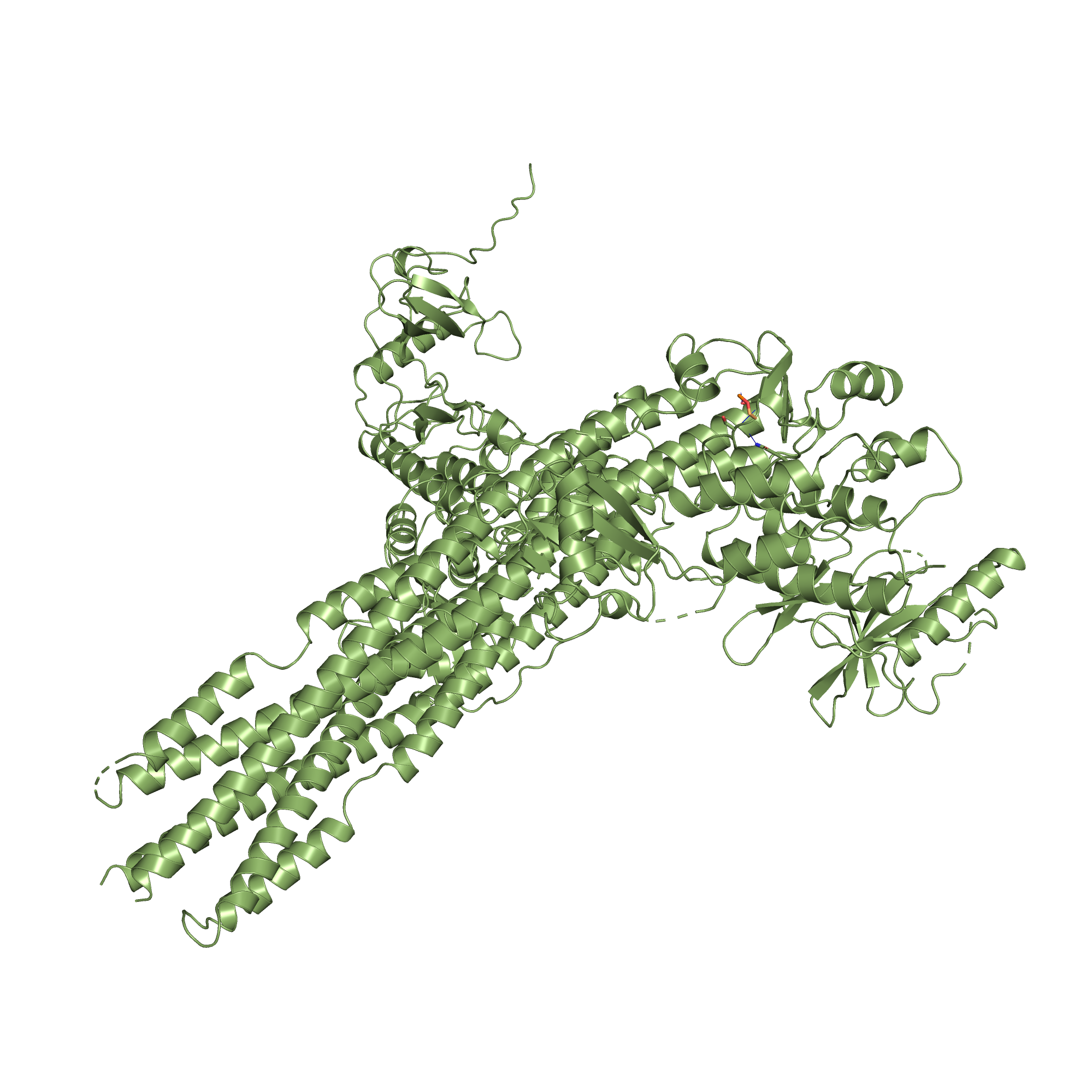

Supplement: Supplementary file 5 [file DataSheet4.ZIP › Figure2 G-I Molecular docking/IkB/ikb/dock2.png]

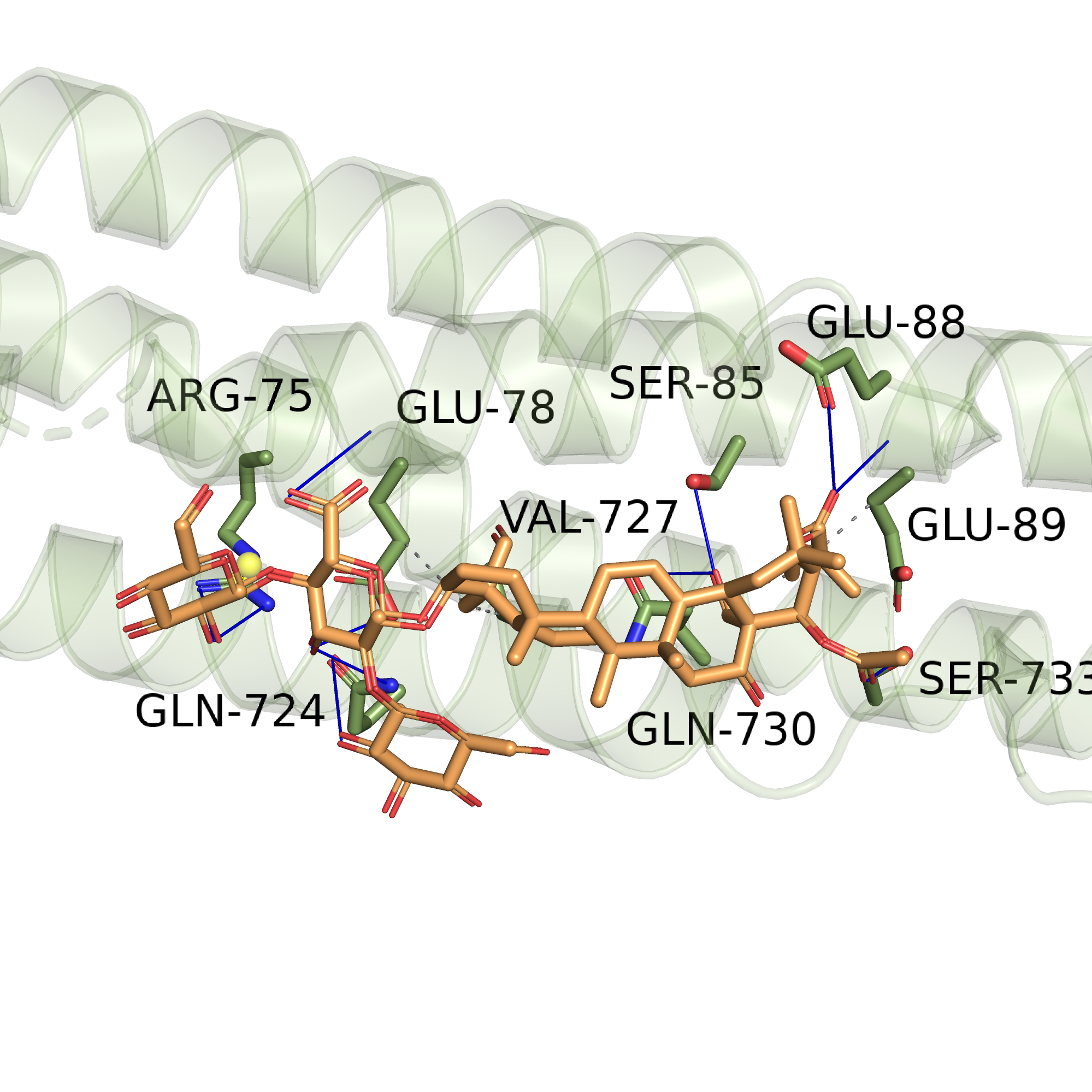

Supplement: Supplementary file 5 [file DataSheet4.ZIP › Figure2 G-I Molecular docking/IkK/dock1.png]

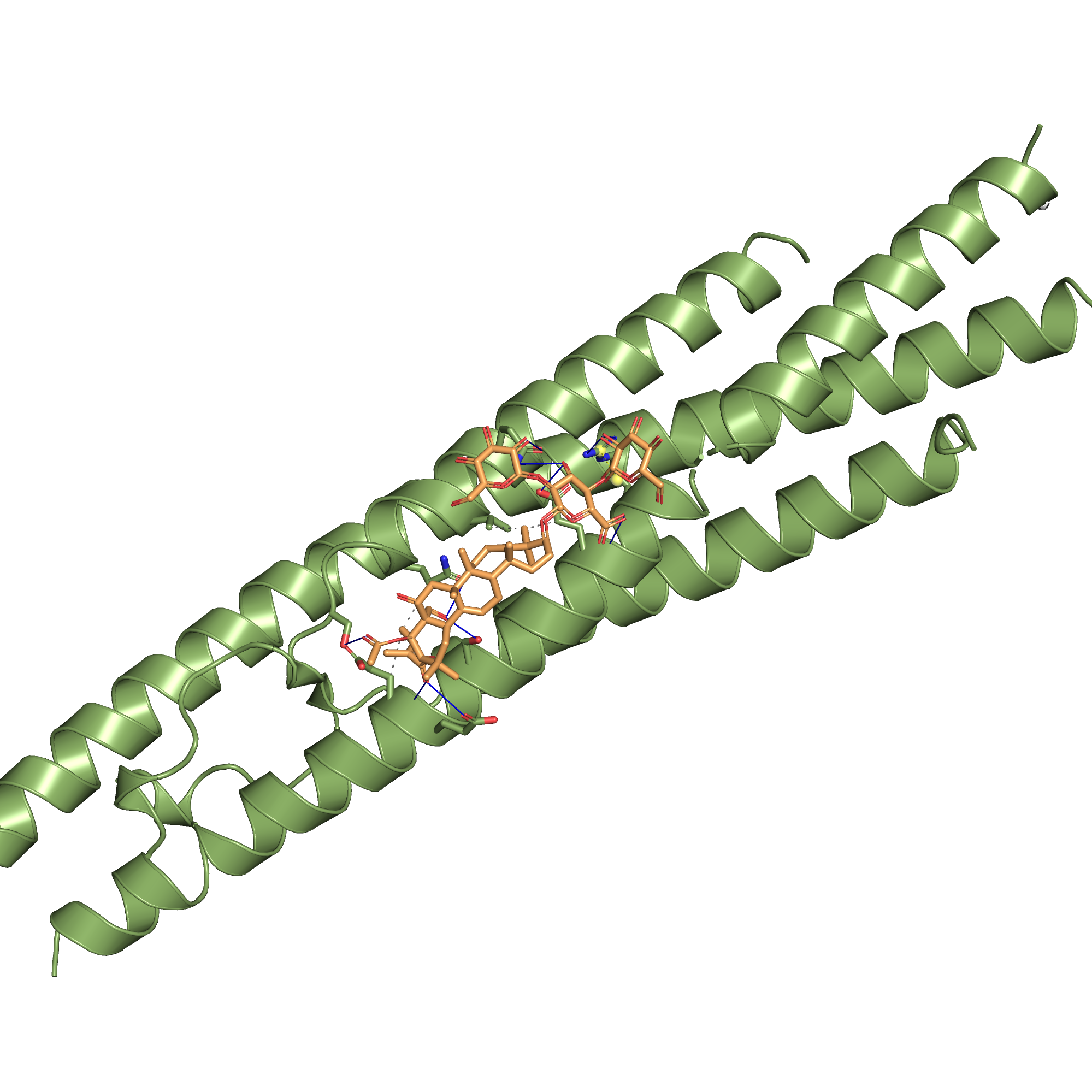

Supplement: Supplementary file 5 [file DataSheet4.ZIP › Figure2 G-I Molecular docking/IkK/dock2.png]

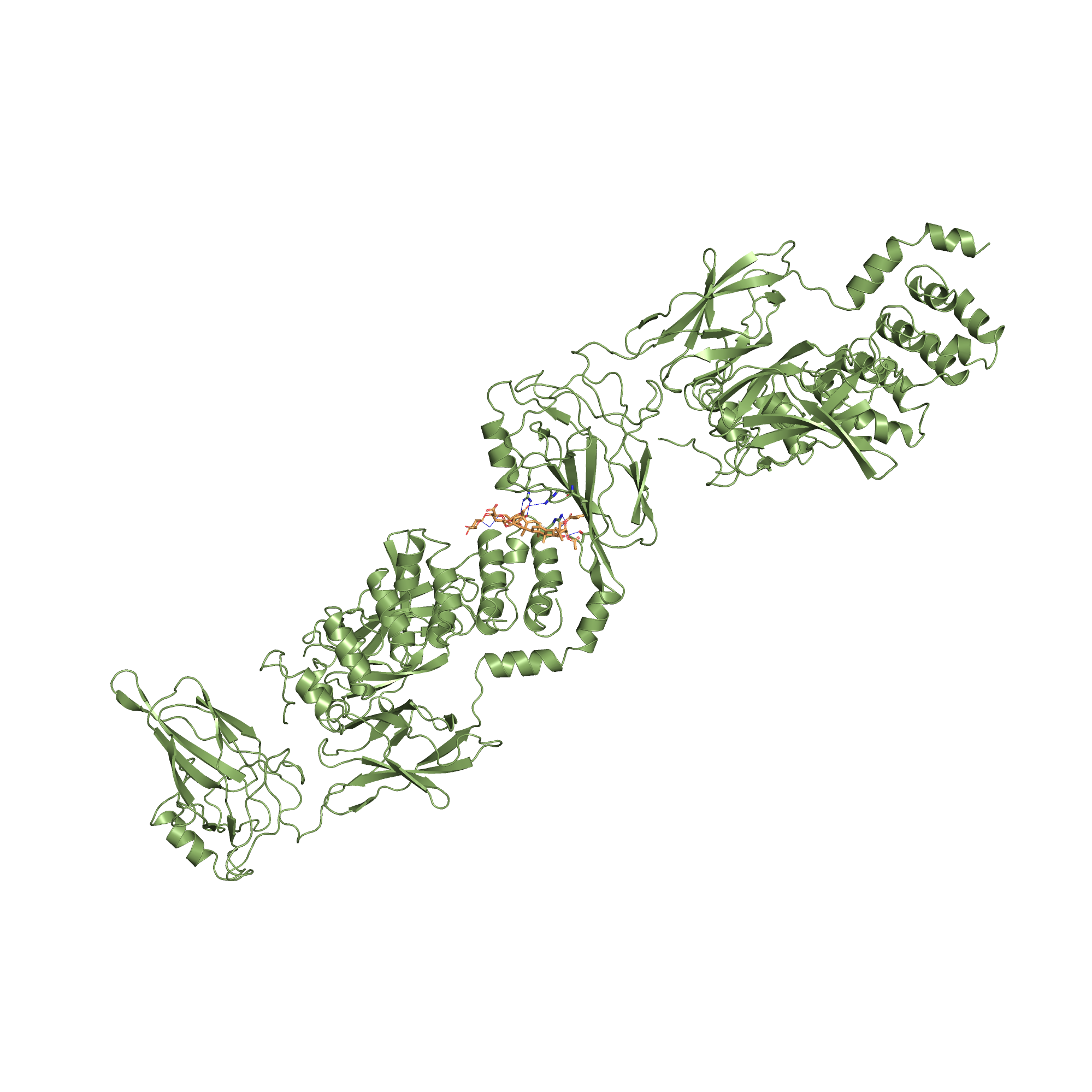

Supplement: Supplementary file 5 [file DataSheet4.ZIP › Figure2 G-I Molecular docking/p65/1efd880f98a7e70bdfd790677aae358.png]

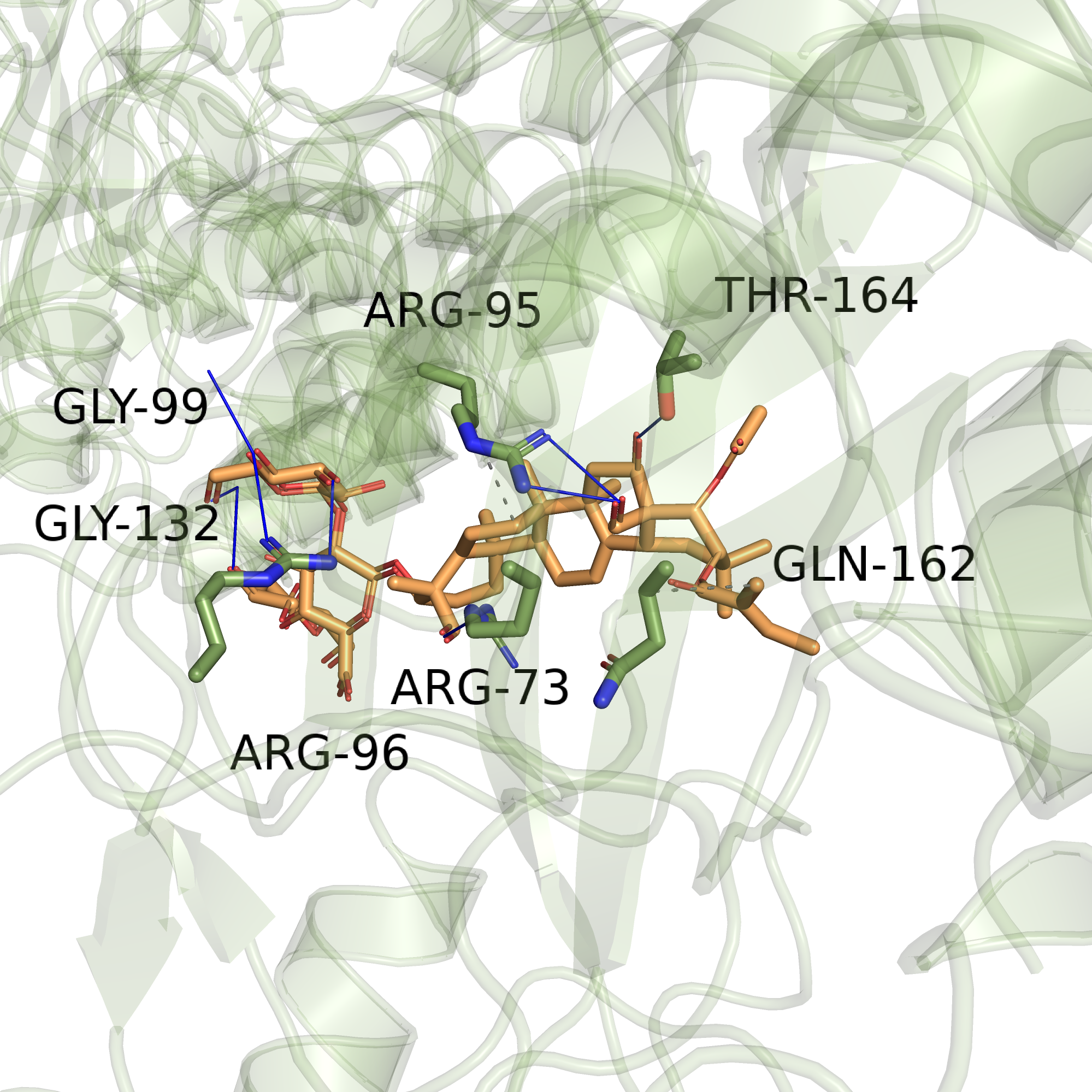

Supplement: Supplementary file 5 [file DataSheet4.ZIP › Figure2 G-I Molecular docking/p65/74aaf0d9bd6c77ca3f6665efb0879be.png]

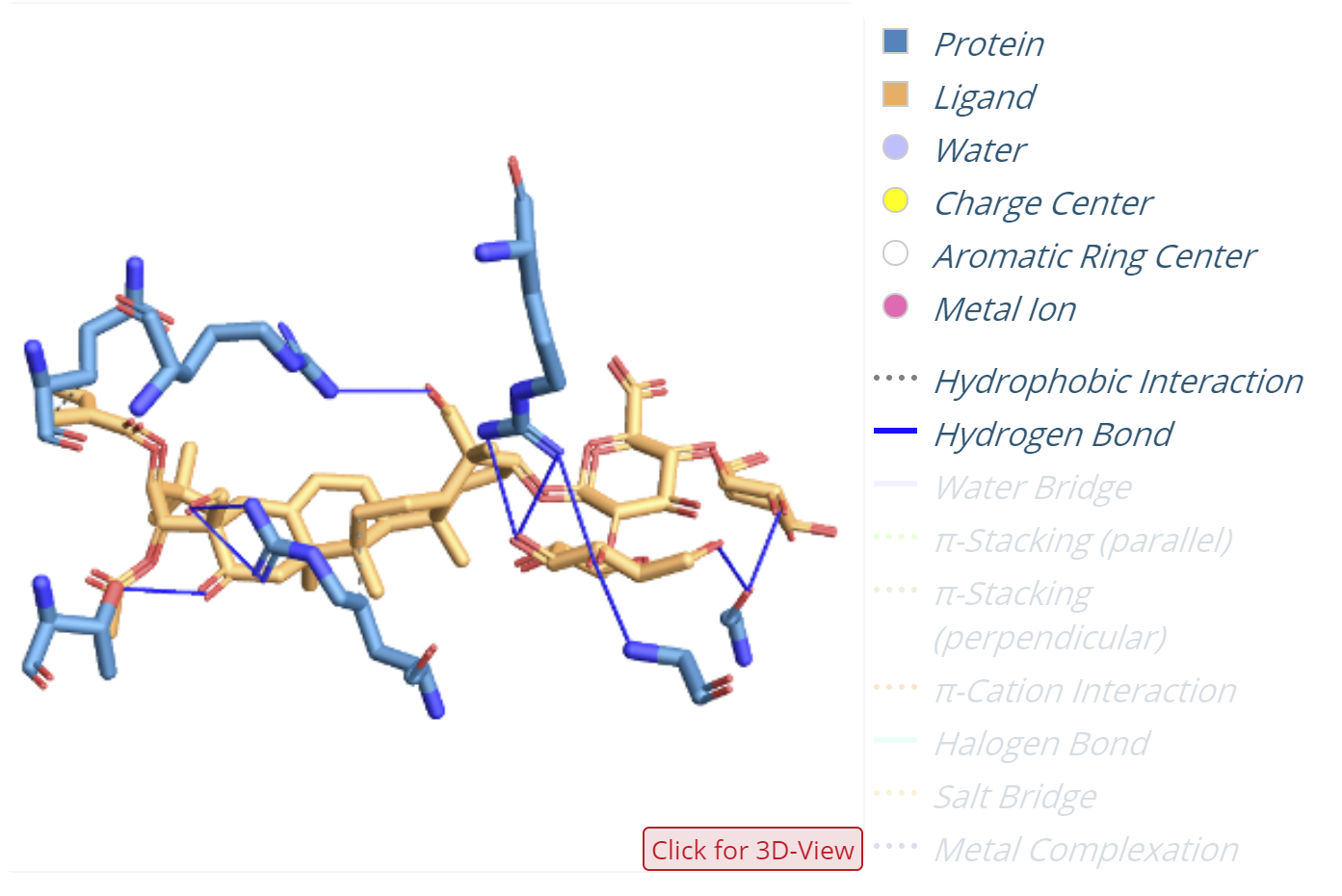

Supplement: Supplementary file 5 [file DataSheet4.ZIP › Figure2 G-I Molecular docking/p65/b77d1938883273771408c2780360e4e.png]

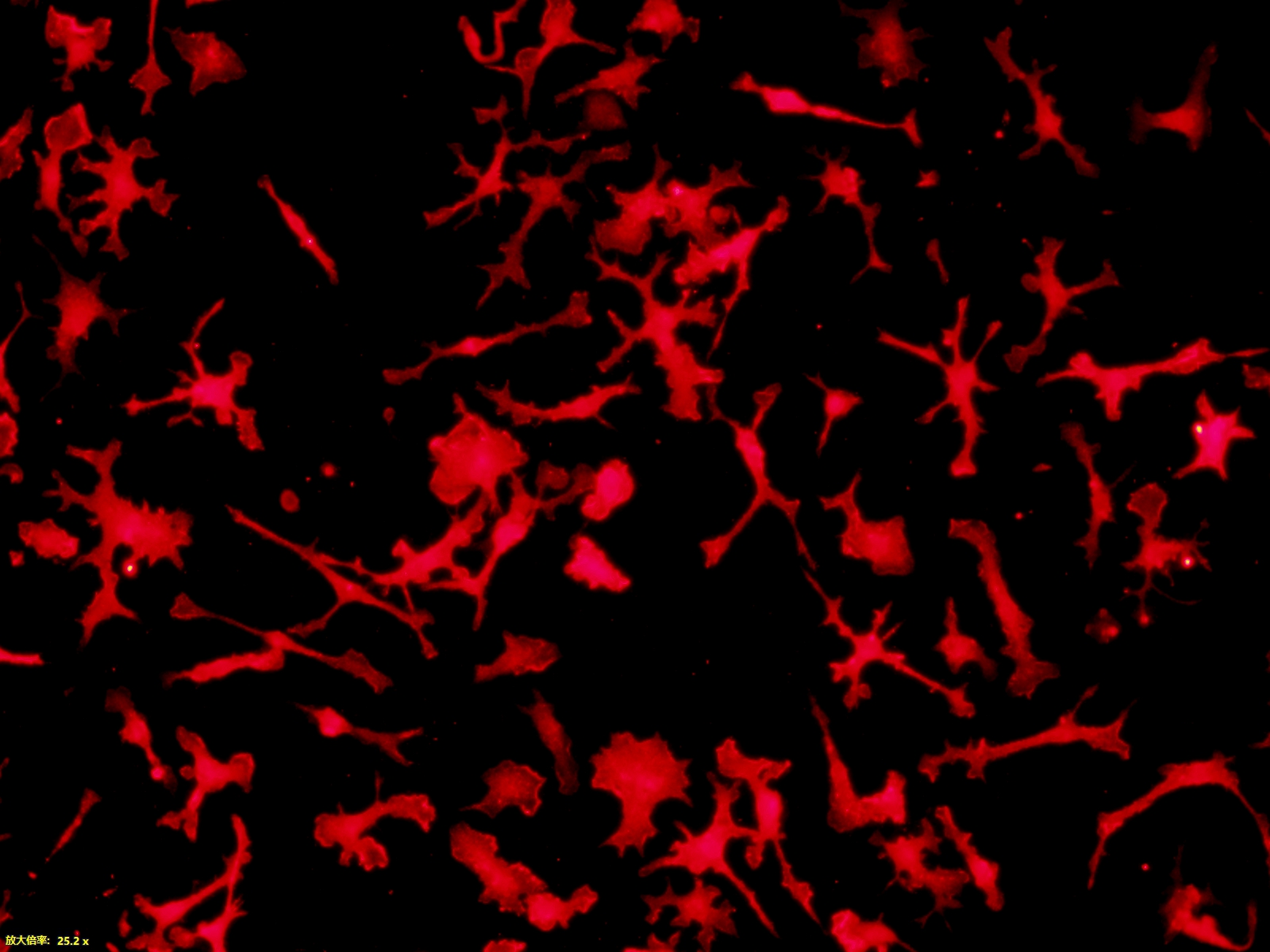

Supplement: Supplementary file 6 [file DataSheet1.ZIP › Figure 3A/═╝╧±_6074.jpg]

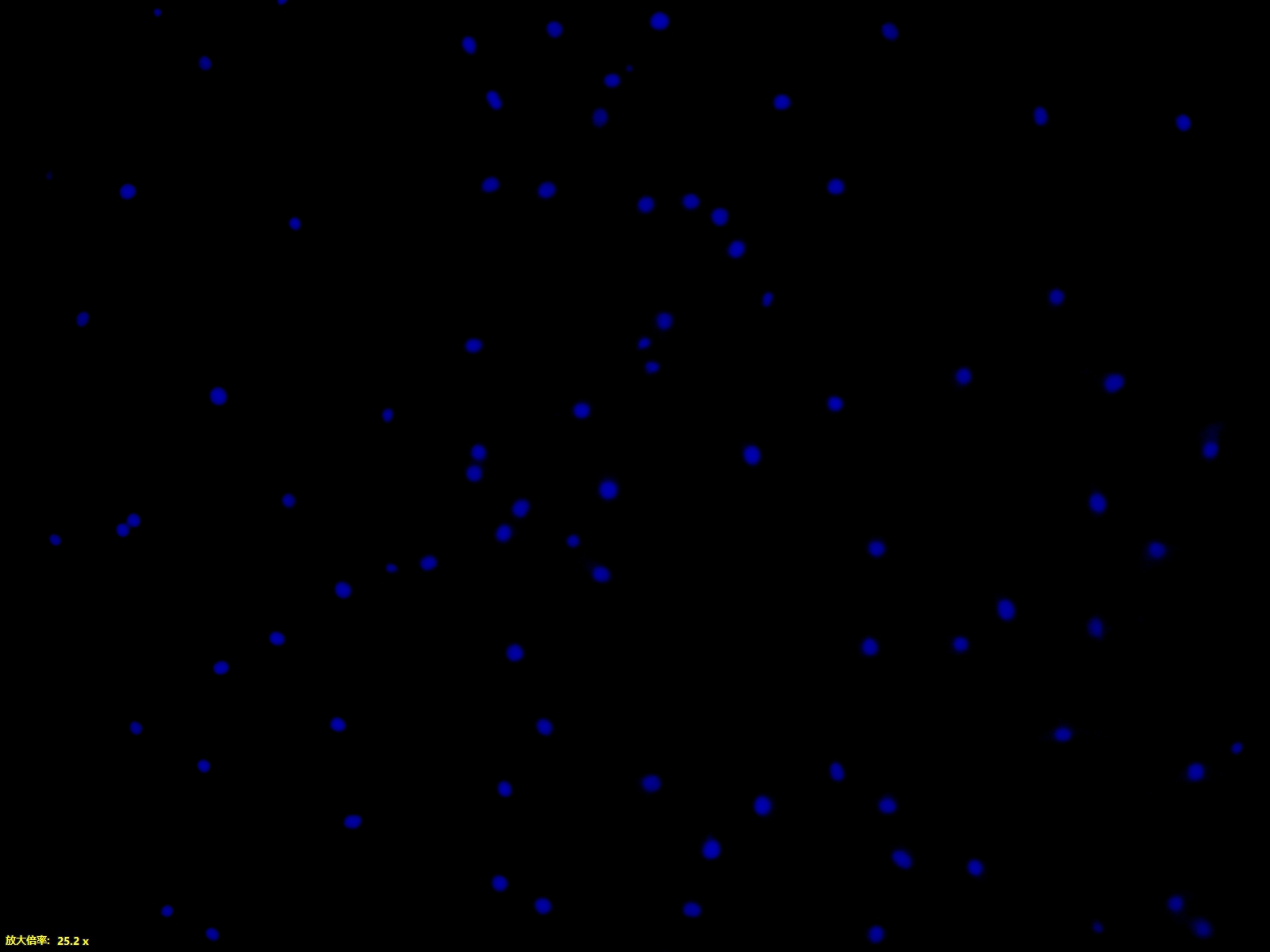

Supplement: Supplementary file 6 [file DataSheet1.ZIP › Figure 3A/═╝╧±_6075.jpg]

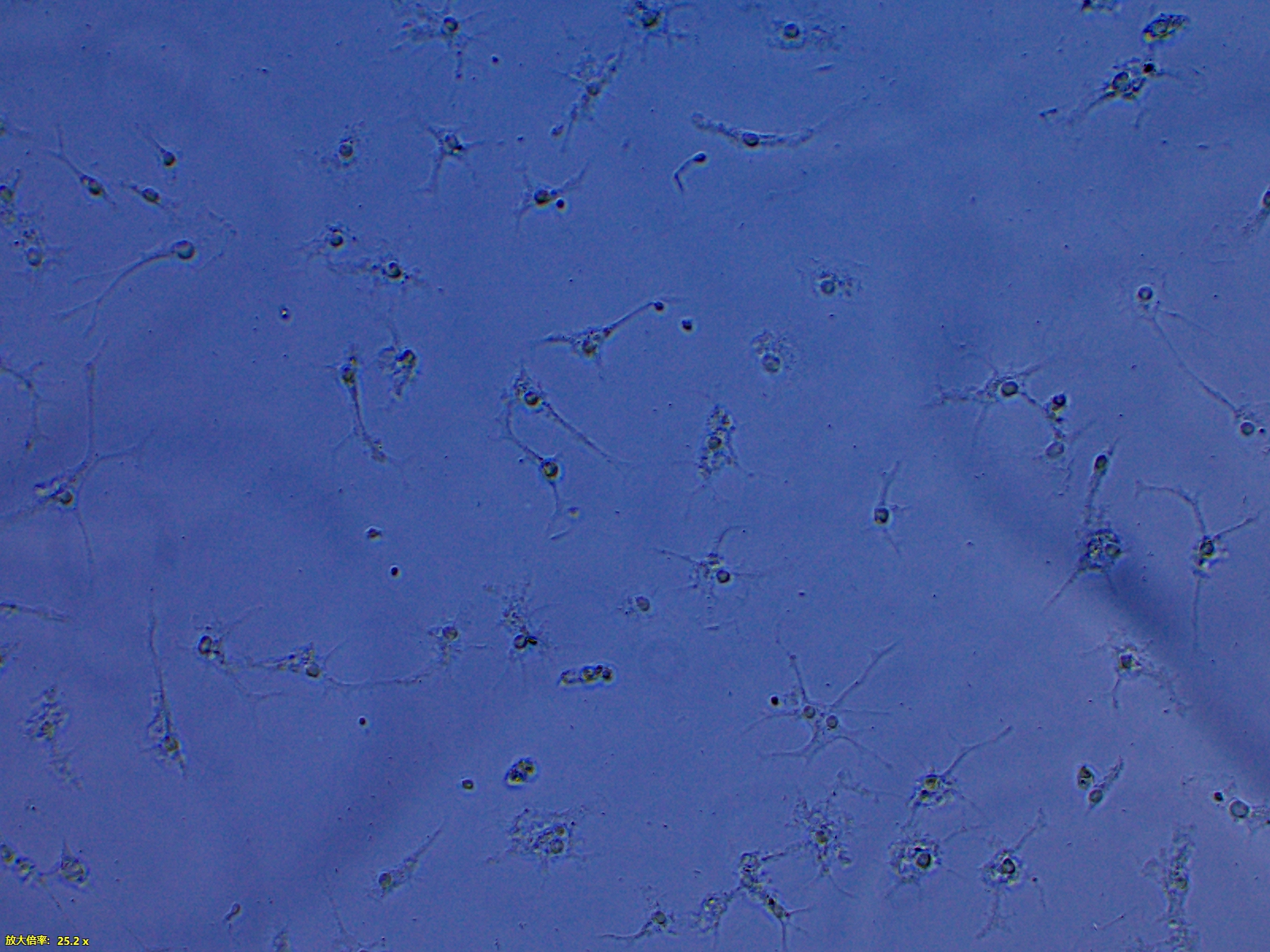

Supplement: Supplementary file 6 [file DataSheet1.ZIP › Figure 3A/═╝╧±_6090.jpg]

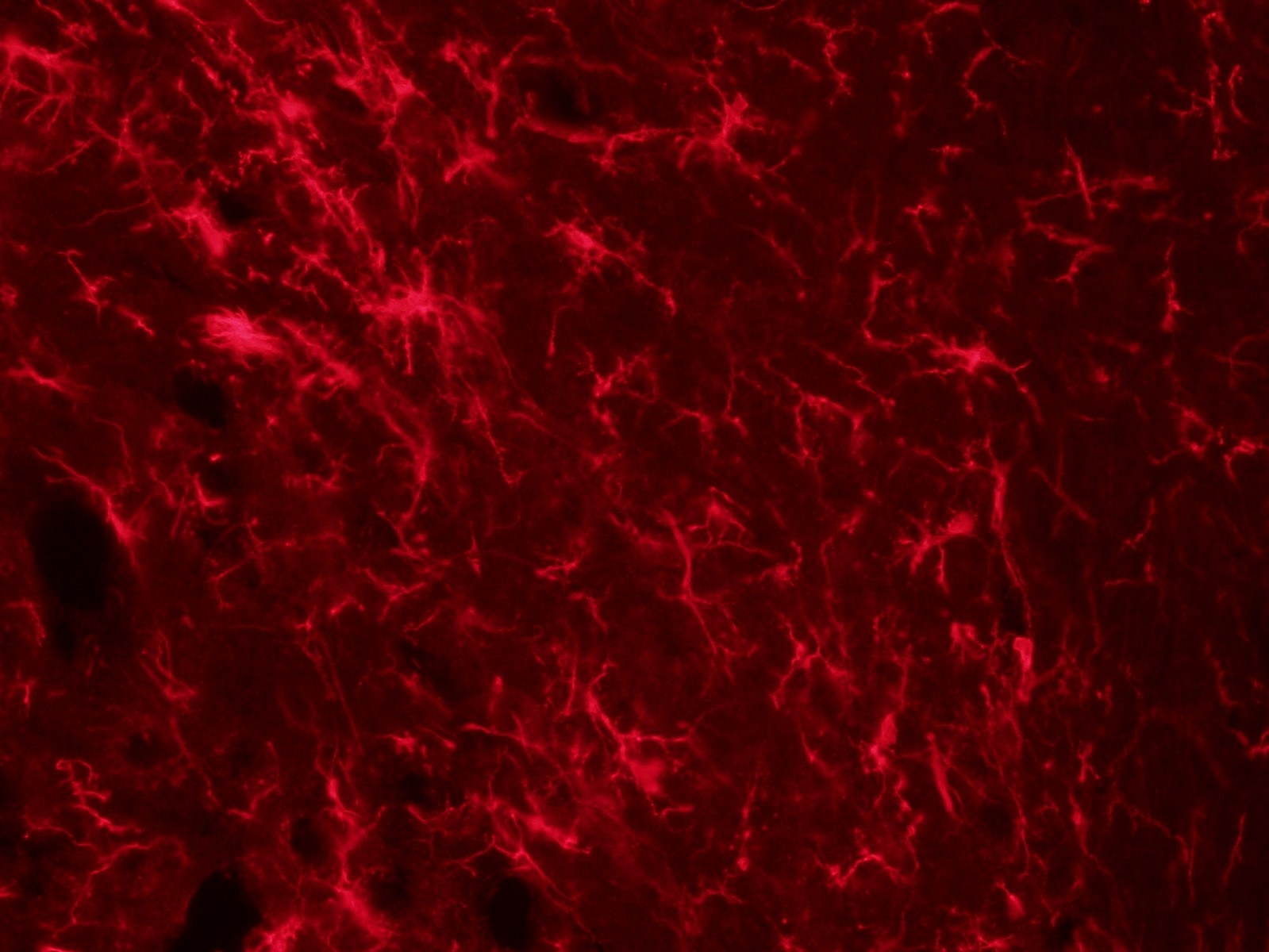

Supplement: Supplementary file 8 [file DataSheet2.ZIP › Figure 4A/LPS+SA.jpg]

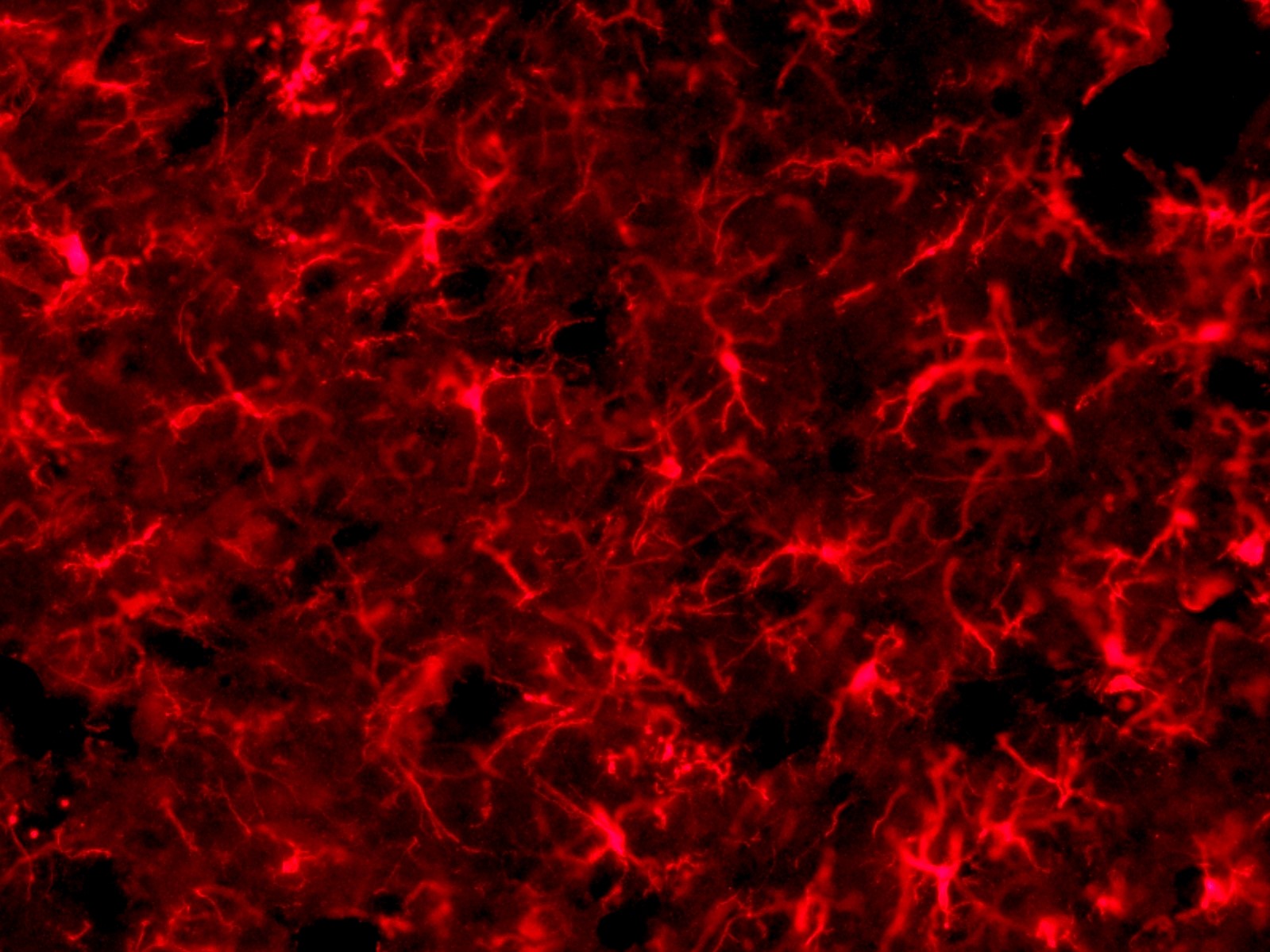

Supplement: Supplementary file 8 [file DataSheet2.ZIP › Figure 4A/LPS.jpg]

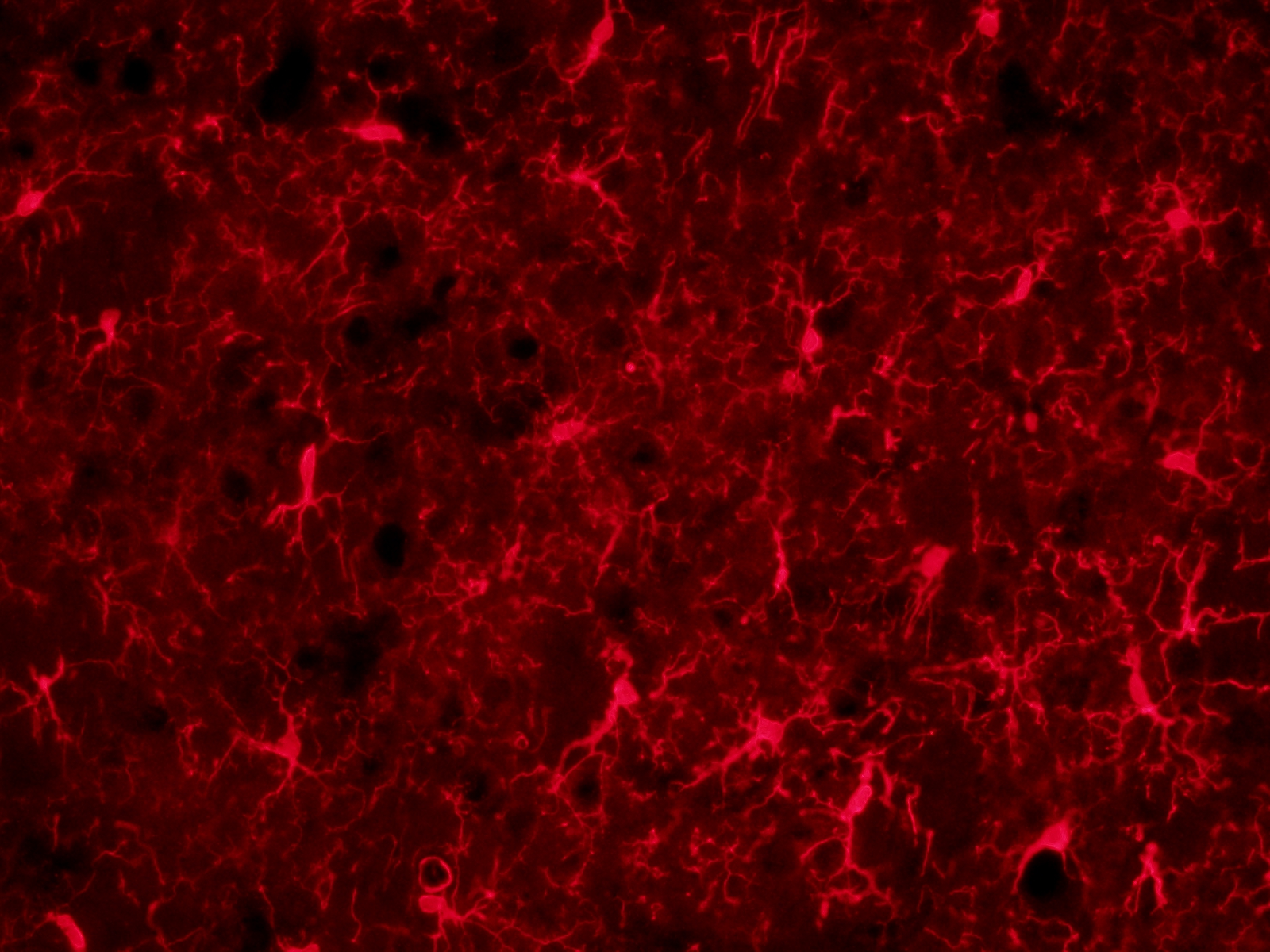

Supplement: Supplementary file 8 [file DataSheet2.ZIP › Figure 4A/Sham 40▒╢-2.jpg]

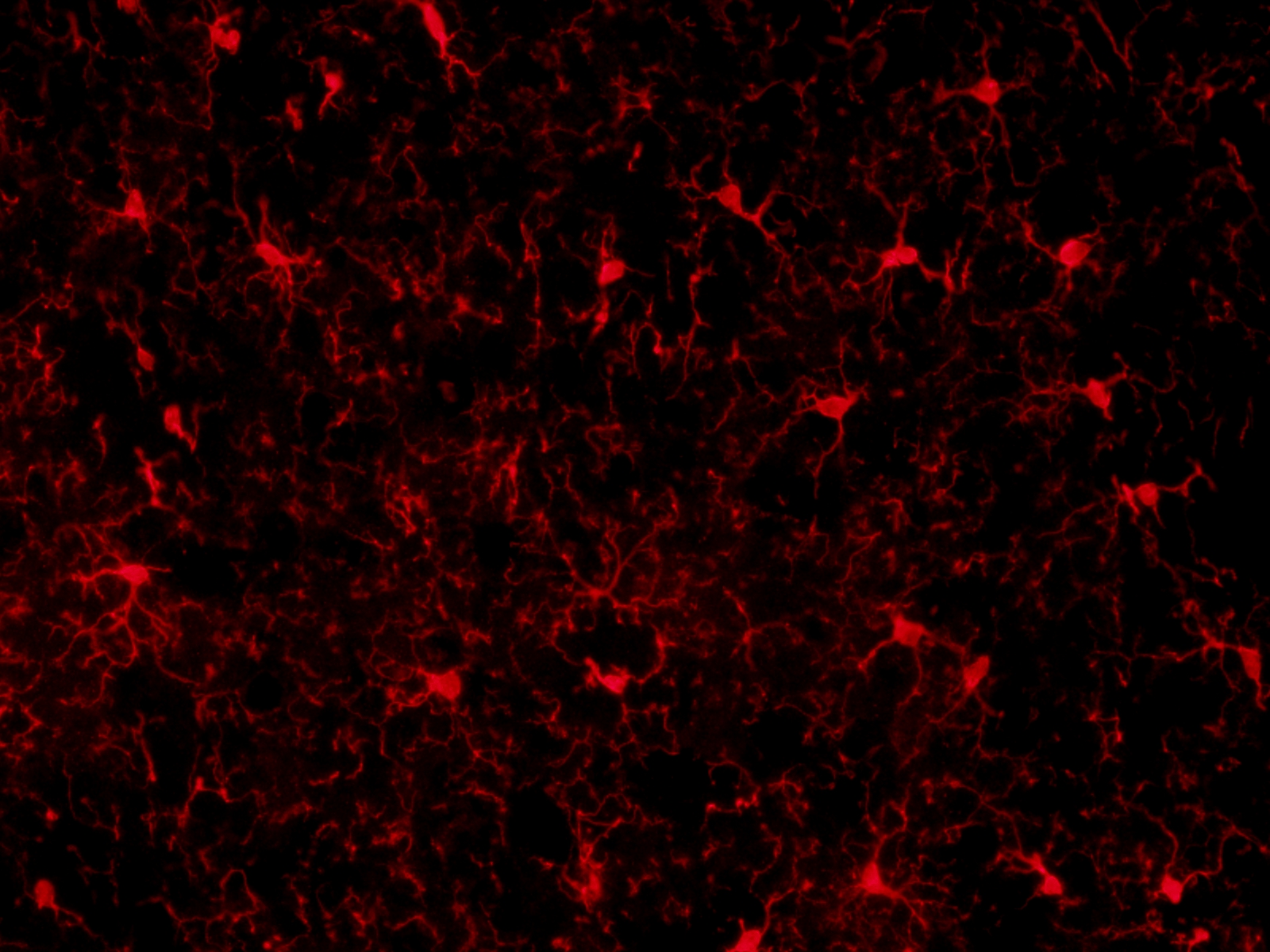

Supplement: Supplementary file 8 [file DataSheet2.ZIP › Figure 4A/Sham+SA 40-4.jpg]
