# Supplementary material for: Sodium aescinate inhibits microglia activation through NF-κB pathway and exerts neuroprotective effect
Source: Front Pharmacol. 2023 Jan 26;14:1086429. doi: 10.3389/fphar.2023.1086429 (PMC9908748; doi:10.3389/fphar.2023.1086429)
Supplement: Supplementary file 4 [file Presentation1.PPTX]

## Slide 1
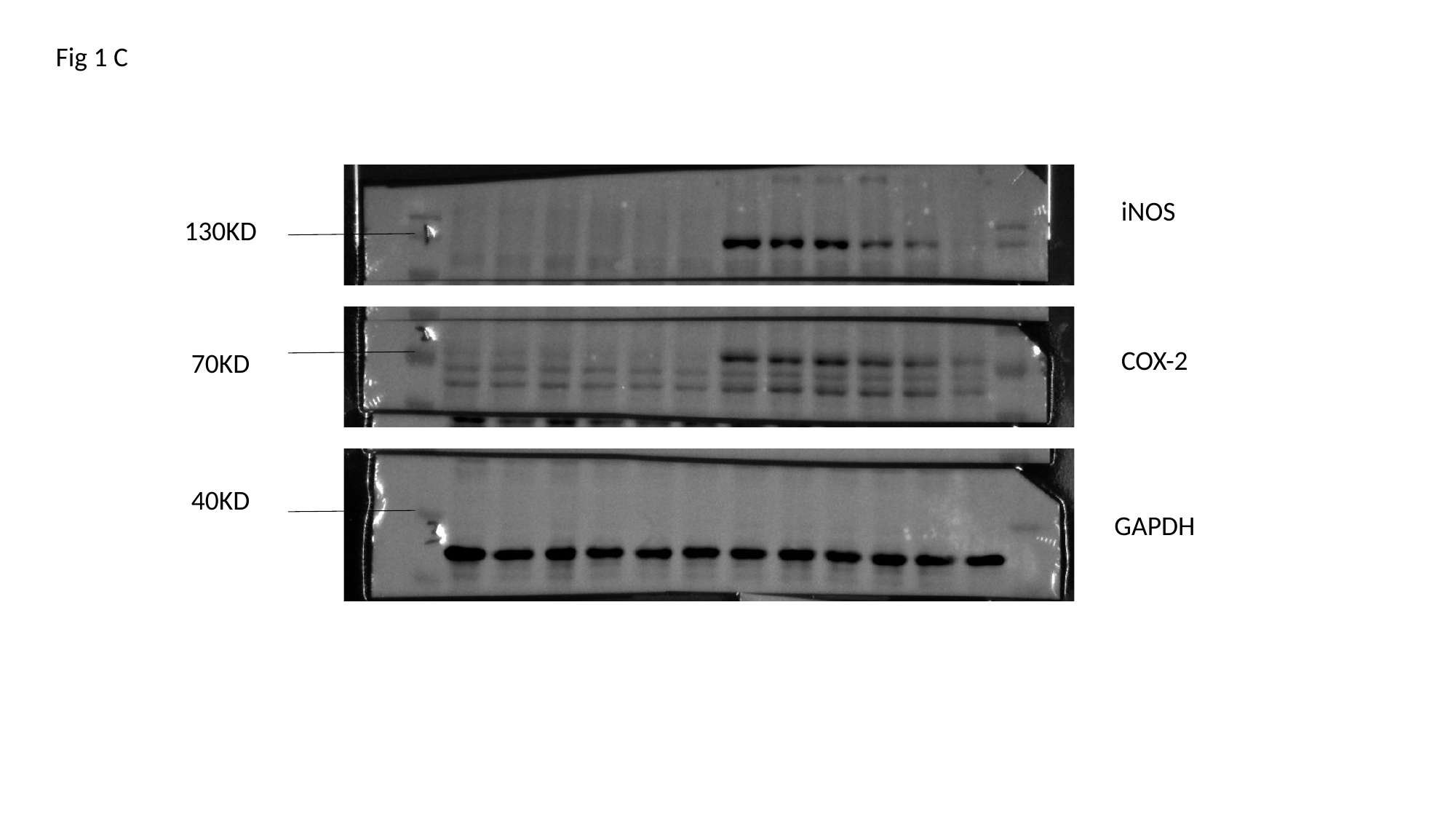

Fig 1 C
iNOS
130KD
COX-2
70KD
40KD
GAPDH

## Slide 2
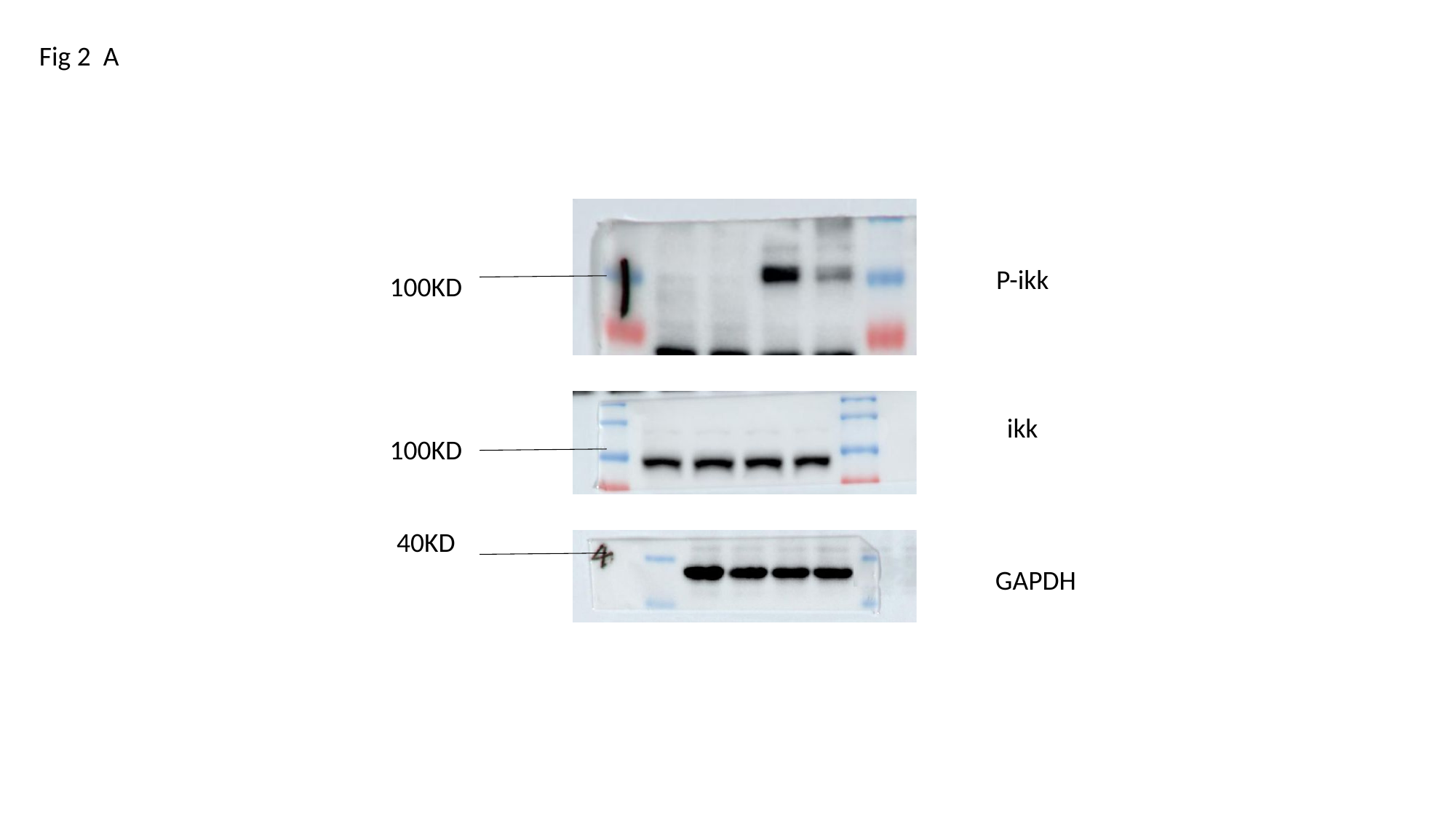

Fig 2 A
P-ikk
100KD
ikk
100KD
40KD
GAPDH

## Slide 3
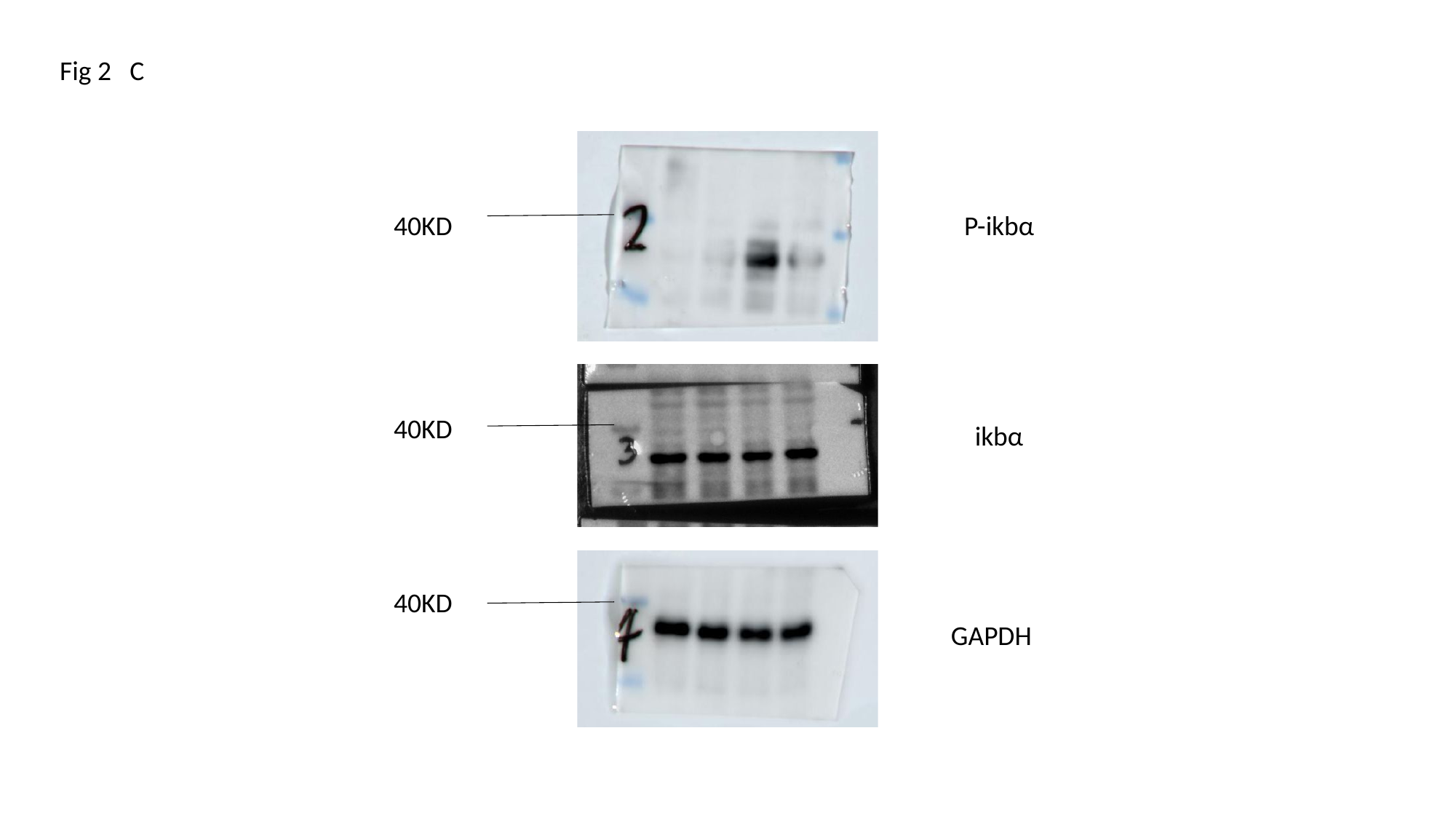

Fig 2 C
40KD
P-ikbα
40KD
ikbα
40KD
GAPDH

## Slide 4
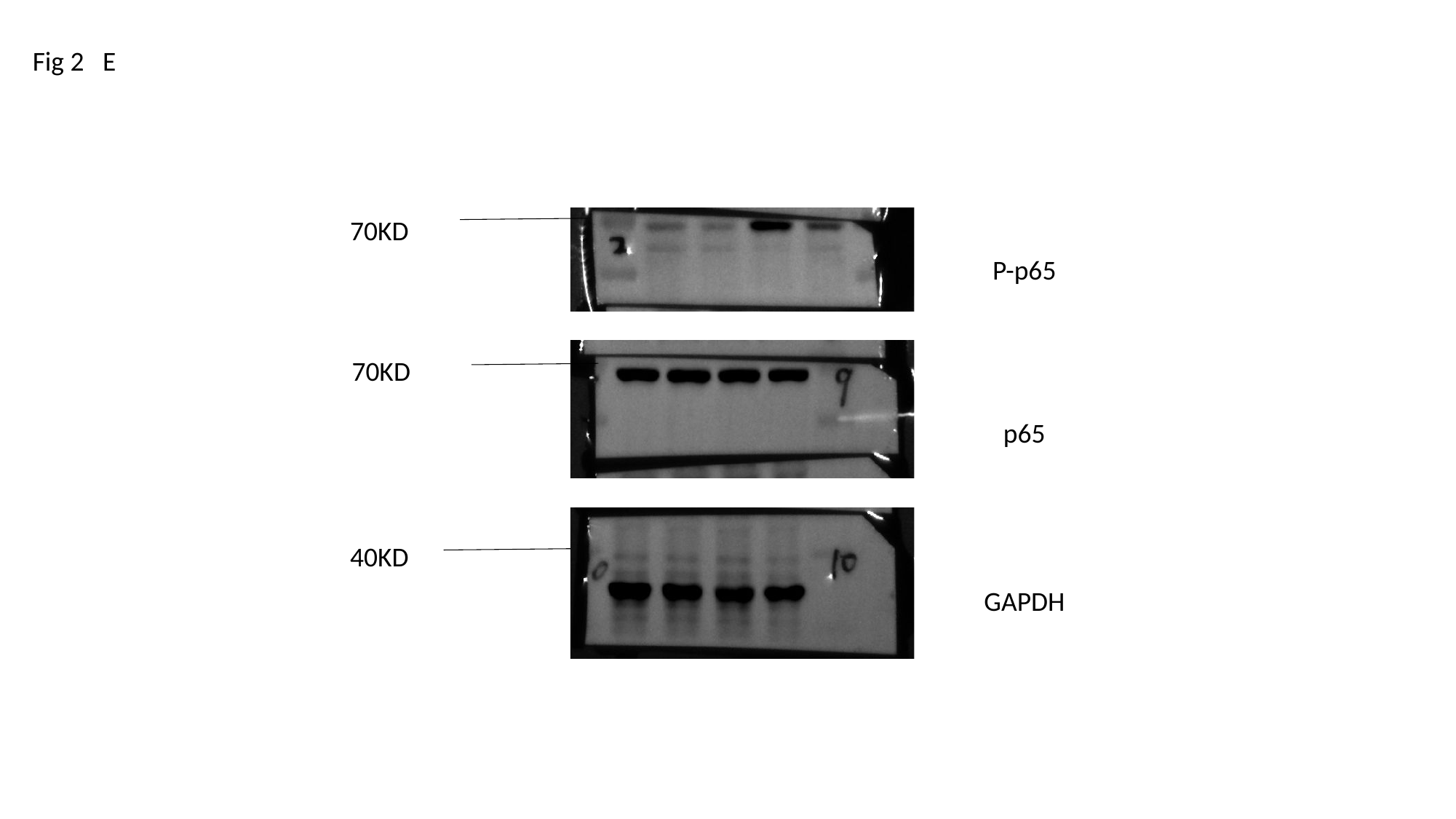

Fig 2 E
70KD
P-p65
70KD
p65
40KD
GAPDH

## Slide 5
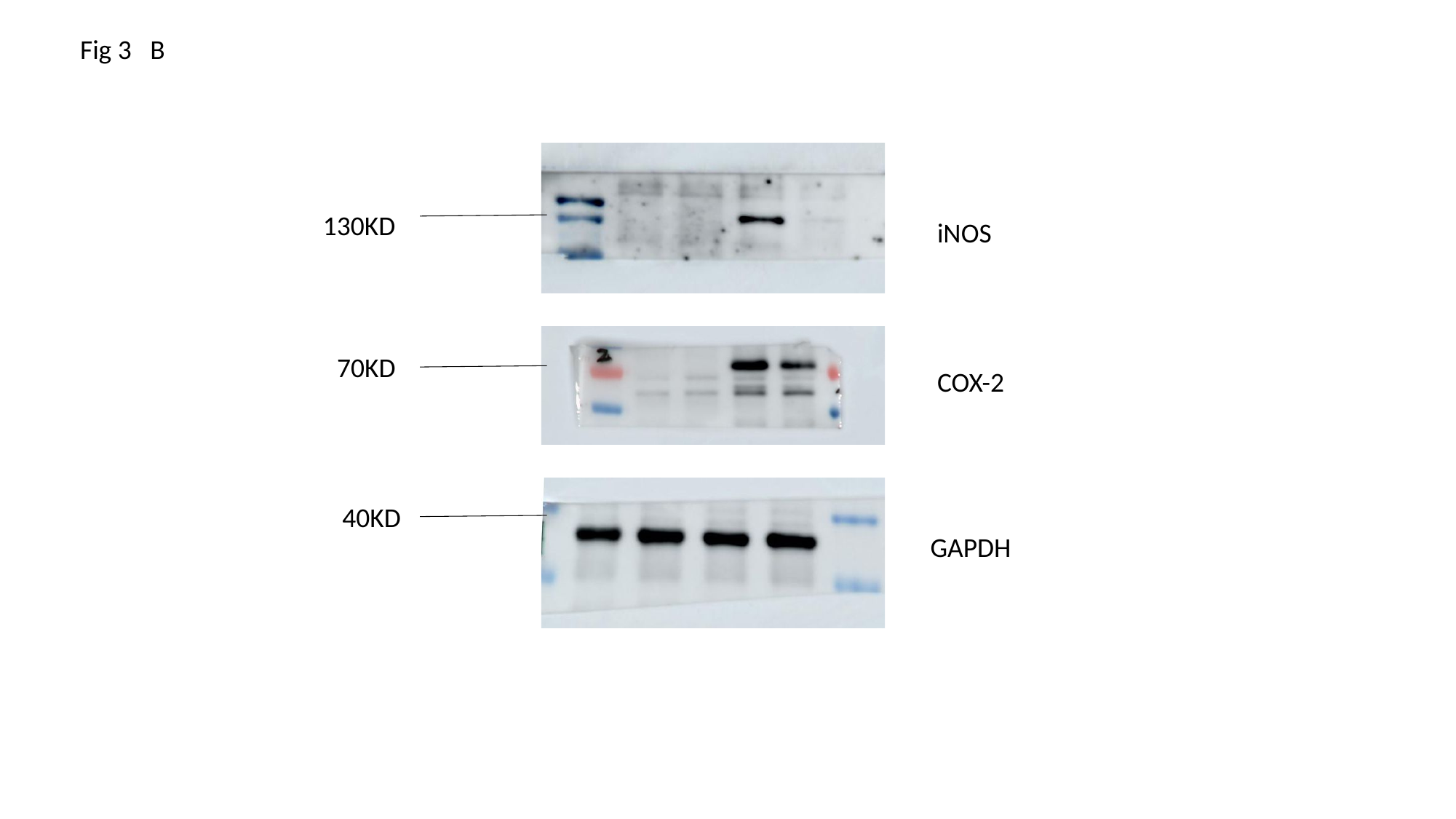

Fig 3 B
130KD
iNOS
70KD
COX-2
40KD
GAPDH

## Slide 6
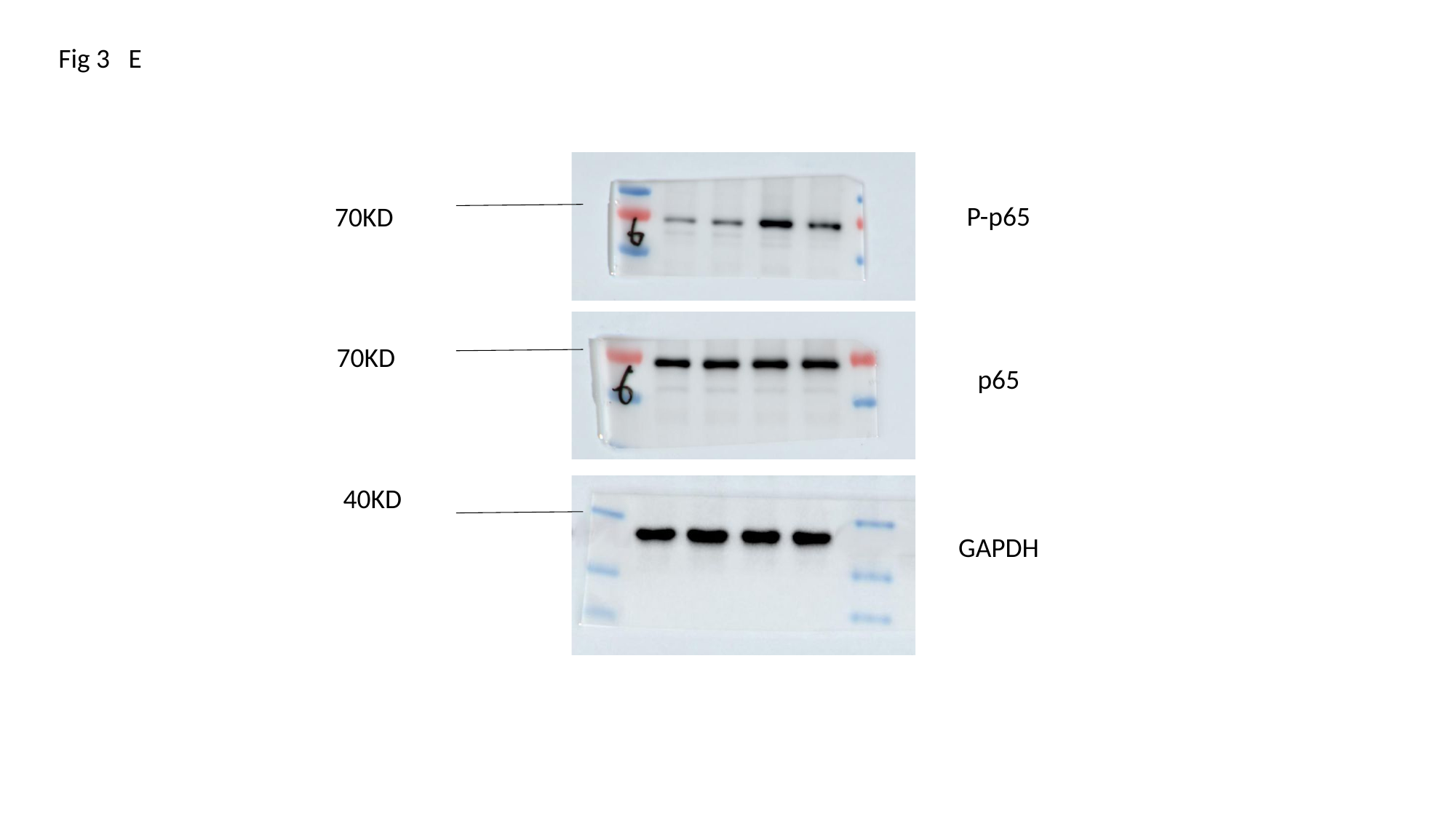

Fig 3 E
P-p65
70KD
70KD
p65
40KD
GAPDH

## Slide 7
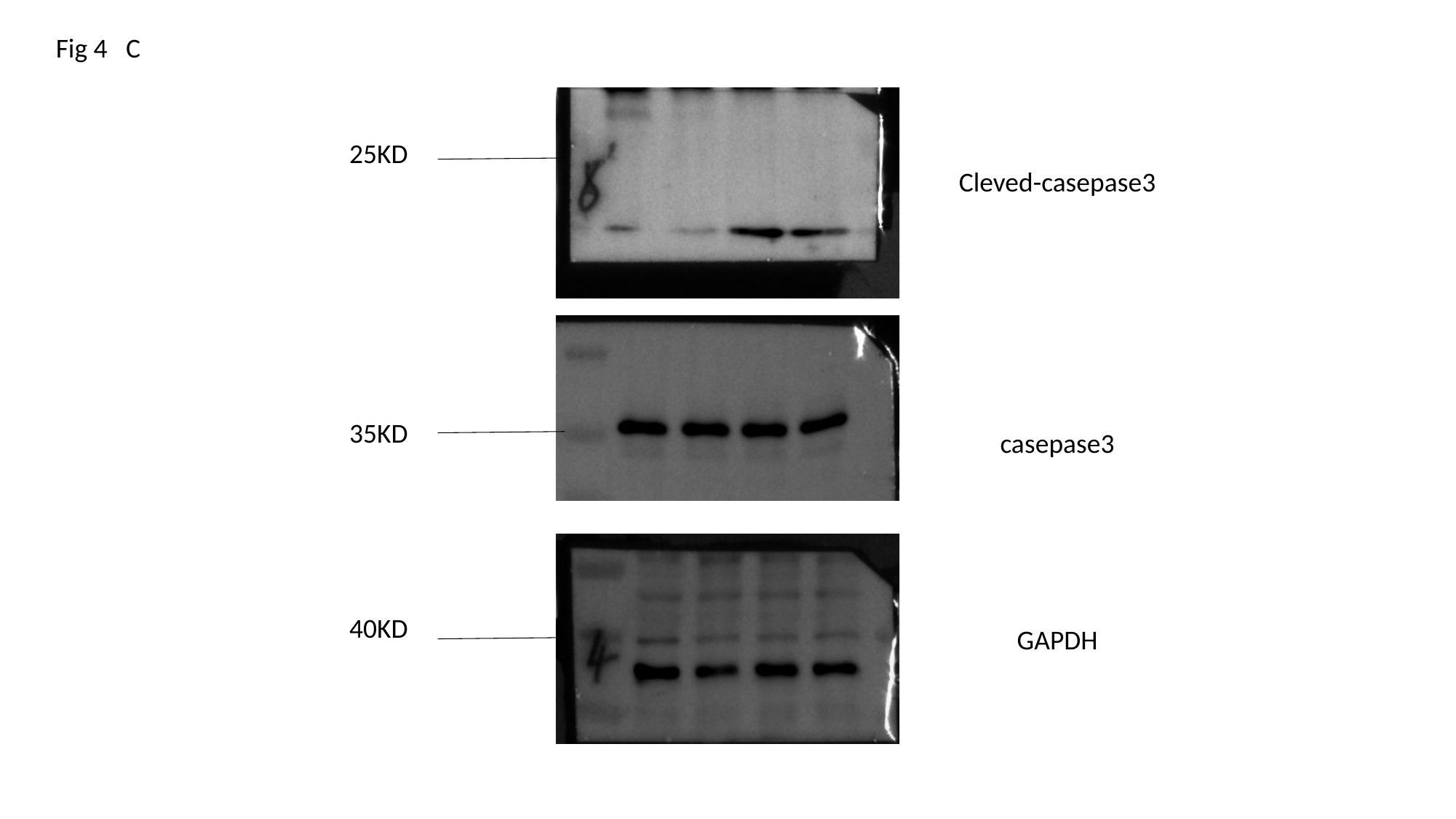

Fig 4 C
25KD
Cleved-casepase3
35KD
casepase3
40KD
GAPDH

## Slide 8
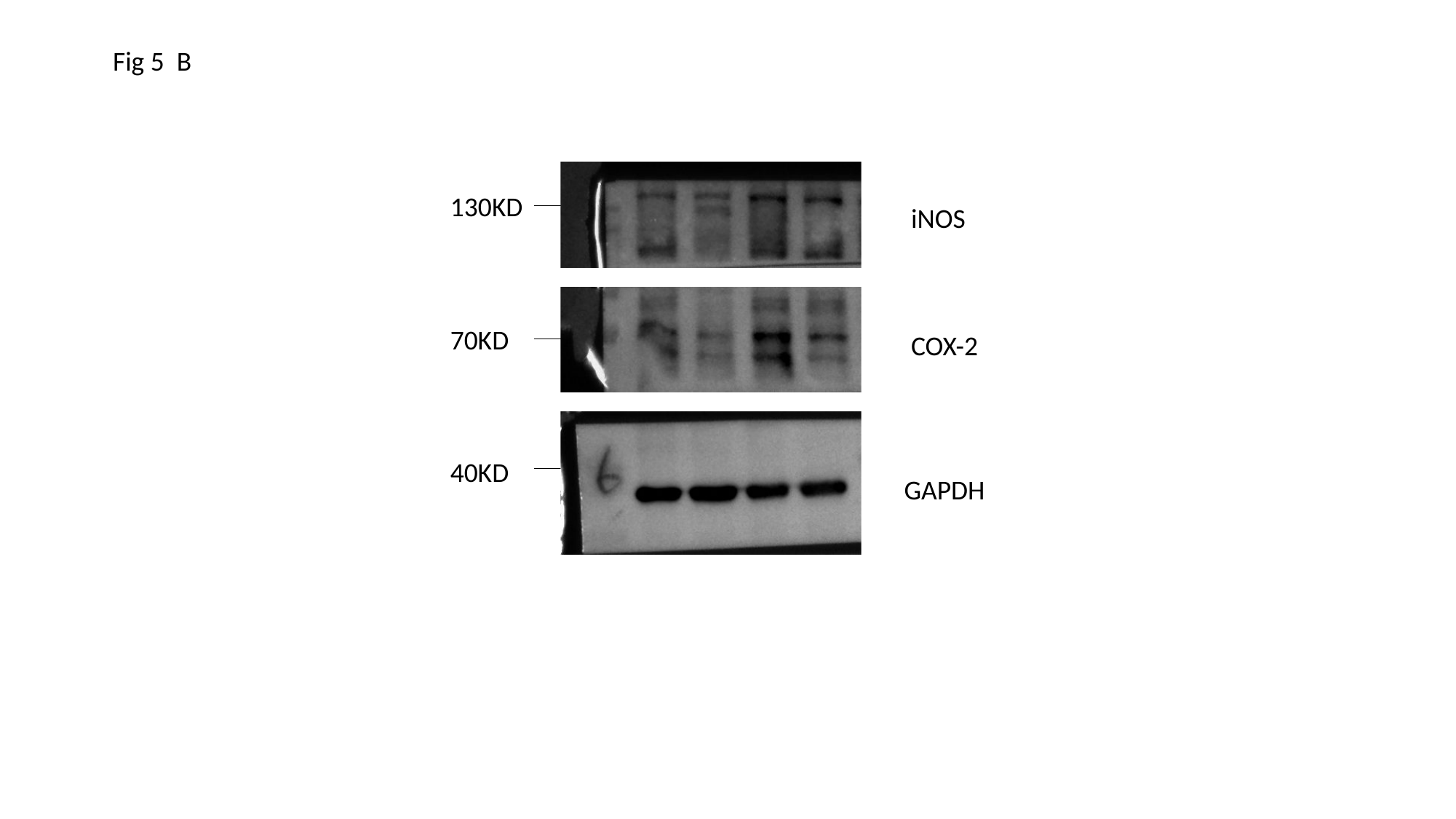

Fig 5 B
130KD
iNOS
70KD
COX-2
40KD
GAPDH
